# Patient‐Derived Bladder Cancer Organoids as a Valuable Tool for Understanding Tumor Biology and Developing Personalized Treatment

**DOI:** 10.1002/advs.202414558

**Published:** 2025-02-07

**Authors:** Hongda Zhao, Na Lin, Vincy Wing Sze Ho, Kang Liu, Xuan Chen, Hongwei Wu, Peter Ka‐Fung Chiu, Linda Huang, Zahra Dantes, Ka‐Leung Wong, Ho‐Fai Chau, Ivan Ching‐Ho Ko, Chris Ho‐Ming Wong, David Ka‐Wai Leung, Steffi Kar‐Kei Yuen, Dinglan Wu, Xiaofan Ding, Chi Fai Ng, Jeremy Yuen‐Chun Teoh

**Affiliations:** ^1^ S.H. Ho Urology Centre Department of Surgery The Chinese University of Hong Kong Hong Kong 999077 China; ^2^ Li Ka Shing Institute of Health Sciences The Chinese University of Hong Kong Hong Kong 999077 China; ^3^ Department of Biomedical Sciences Faculty of Health Sciences University of Macau Taipa Macao SAR 999078 China; ^4^ Invitrocue Hong Kong Ltd Hong Kong SAR 999077 China; ^5^ Invitrocue Europe AG 80539 Munich Germany; ^6^ Department of Applied Biology and Chemical Technology The Hong Kong Polytechnic University Hong Kong 999077 China; ^7^ Department of Urology Medical University of Vienna Vienna 1090 Austria

**Keywords:** bladder cancer, drug screening, organoids, personalized medicine, tumor microenvironment

## Abstract

Bladder cancer (BC) is a heterogeneous disease with high recurrence rates and variable treatment responses. To address these clinical challenges, the world's first bladder cancer patient‐derived organoids (PDOs) biobank is established based on an Asian population. Thirty‐six BC‐PDOs are generated from 56 patients and demonstrated that the BC‐PDOs can replicate the histological and genomic features of parental tumors. Drug screening tests are conducted with a broad spectrum of conventional chemotherapeutic and targeted therapy drugs and identified differential drug sensitivities among the BC‐PDOs. These in vitro results are consistently supported by the PDO xenograft animal studies and patients’ clinical treatment outcomes, thereby verifying the predictive power of PDOs for drug responses in BC patients. By analyzing the genetic profiles of the PDOs, specific driver genes that correlate with drug sensitivity to two stand‐of‐care chemotherapeutics, cisplatin, and gemcitabine, are identified. Additionally, the practicality of PDOs in investigating the tumor microenvironment has been demonstrated. This study underscores the utility of PDOs in advancing the understanding of bladder cancer and the development of personalized therapeutic strategies. The BC‐PDOs biobank provides an ideal preclinical platform for supporting the development of personalized treatment strategies for BC patients. This study also provides insights into the potential mechanisms of drug resistance, paves the way for subsequent region‐specific research, and demonstrates the possibility of using PDO‐related models to direct future research in developing drugs targeting tumor microenvironments.

## Graphical abstract

We have established the first Asian‐based bladder cancer PDOs biobank, demonstrating their accuracy in mirroring parental tumors’ properties. Drug screening reveals diverse sensitivities to chemotherapies and targeted therapies, validated by PDO xenografts and patient outcomes. Genetic profiling identifies key genes associated with response to standard chemotherapies. Co‐culture model highlights the potential of PDOs in exploring the tumor microenvironment.

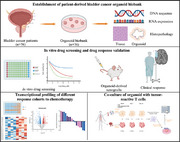

## 1 Introduction

Bladder cancer includes two main disease conditions, non‐muscle invasive bladder cancer (NMIBC) and muscle‐invasive bladder cancer (MIBC). For patients with NMIBC, the standard treatment approaches include transurethral resection of bladder tumor (TURBT) operation, single‐dose intravesical chemotherapy, and Bacillus Calmette‐Guérin (BCG) treatment. However, the oncological outcome is not satisfactory.^[^ ^1^ ^]^ For patients with MIBC, radical cystectomy is the traditional treatment method, but it is associated with significant complications. Some recent genetic studies have highlighted substantial molecular differences between NMIBC and MIBC.^[^ ^2^ ^]^ In addition, these studies indicated that bladder cancer is a heterogeneous cancer with various subtypes and different genomic mutations.^[^ ^3^ ^]^ These analyses suggest a correlation between the genomic profile and the clinical behavior of bladder cancer. For example, patients with TP53 and ERBB2 (HER2) mutations have a higher risk of tumor recurrence and progression.^[^ ^4^ ^]^ Furthermore, significant disparities are observed among different racial groups, such as the variations in survival outcomes and genomic profiles.^[^ ^5^ ^]^ To comprehensively understand the biological implications of common mutations, conduct region‐specific research, and facilitate the development of precise, personalized treatment methods, it is imperative to establish dependable personalized models that closely resemble the original tumor.

Recently, the scientific community has focused on using PDOs, a 3D in vitro model, as an experimental approach for developing precision medicine. The tumor cells were isolated from parental tissues and effectively cultivated in the form of PDOs, accurately reflecting their structure and heterogeneity while preserving genetic integrity in vitro.^[^ ^6^ ^]^ Although initial work has established the feasibility of culturing bladder cancer‐derived PDOs, a thorough description of their biological characteristics, including genomic mutations and transcriptional profiles, is yet to be fully elucidated.^[^ ^7^ ^]^ In addition, reports on the application of BC PDOs remain scarce, withlimited data on drug screening, in vivo experimentation, and the exploration of tumor microenvironment. To advance the field, there is a clear need for more extensive research to assess the potential of PDOs in personalized treatment.

In this study, we have successfully established the world's first PDOs biobank for bladder cancer based on an Asian population, which effectively replicates the histological and genomic features of parental tumors. Utilizing these PDOs, we conducted a drug screening process to evaluate the sensitivity to various chemotherapeutic targeted drugs. The responses observed were subsequently validated by in vivo experiments. By analyzing the molecular profiles of PDOs and their parental tumors, we identified specific driver genes that correlate with the sensitivity to certain chemotherapy drugs. Furthermore, we innovatively used PDOs to investigate the interaction between tumor cells and tumor‐reactive T cells. This study underscores the importance of PDOs in enhancing our understanding of tumor biology, facilitating region‐specific research, improving clinical practice, and developing personalized therapeutic strategies. The establishment of PDO biobank provides a robust platform for future research.

## 2 Results

### 2.1 Establishment of a Living BC‐PDOs Biobank

**Figure 1:**
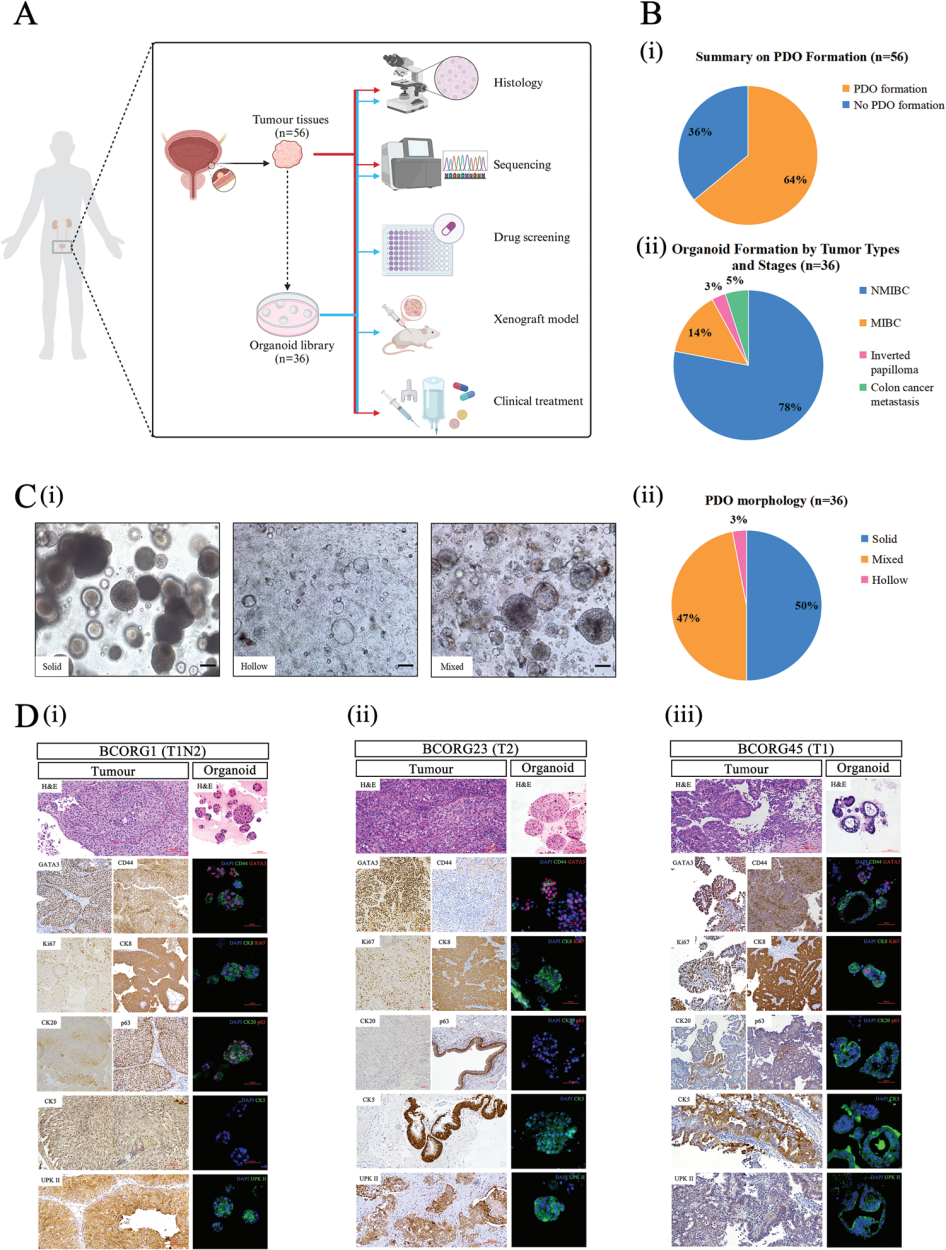
Establishment of BC‐PDOs biobank. A) Schematic of experimental design. Fresh tumor tissues were collected from patients with bladder cancer and processed as described in the methods. Red lines and arrows indicate experiments done on tumor tissues, while blue lines and arrows indicate experiments done with PDOs. (Created in BioRender. Ho, V. (2024) https://BioRender.com/p29z766). B) Summary of PDO formations. (i) Summary of PDO formation among all collected samples. (ii) Tumor types and stages of the cases forming organoids. C) PDO morphology subtypes. (i) Representative brightfield images of morphology subtypes: Solid, Hollow, and mixed (Scale bars, 100 µm). (ii) Percentage of different subtypes across all PDOs. D) Representative micrographs on parental tumors and matched PDOs. H&E and IHC staining on parental tumors for indicated markers, alongside H&E and whole‐mount IF staining of matched PDOs for indicated markers (i) BCORG1 (ii) BCORG23 (iii) BCORG45. Scale bars: 50 µm, 100 µm.

To establish a living BC PDOs biobank, we recruited 56 patients with bladder tumor and collected fresh tumor specimens to develop organoids (**Figure** **1**A). Among these cases, 35 (62.5%) cases were NMIBC, and 15 (26.8%) cases were MIBC, 2 cases were metastatic adenocarcinoma from colon, 2 cases were adnocarcinoma from prostate, and 2 cases were inverted papilloma, demonstrating that our biobank includes known population‐level variety of histology (Table S1, Supporting Information) Patients’ clinical and pathological characteristics are described in Table S1 (Supporting Information). According to previous publications, BC organoid was defined as a cellular cluster that can self‐organize and self‐renew.^[^ ^7^, ^8^ ^]^ Using the microscope, we found 36 (64%) cases have successfully formed organoid structures in Matrigel (Figure 1B), including 28 NMIBCs, 5 MIBCs, 1 inverted papilloma, and 2 colon cancer metastases. Compared to the MIBC group (5 out of 15, 33.3%), the NMIBC group had a higher success rate (28 out of 35, 80%), possibly due to the tumor purity and neoadjuvant chemotherapy.^[^ ^7c^ ^]^ From our experience, MIBC samples exhibit inferior quality compared to NMIBC, with a higher contamination of stroma or adipose tissue, which compromises tumor purity. This impurity affects the material exchange between tumor cells and culture environment, thereby negatively impacting the growth of tumor cells. In addition, some patients with MIBC would receive preoperative chemotherapy, which could reduce the viability of tumor cells.

### 2.2 PDOs Preserve Morphological and Histological Features of Parental Tumors

To record the morphological features of BC PDOs, we analyzed bright‐field images of PDOs and classified them into three groups based on previous report^[^ ^7c^ ^]^: solid, hollow, or mixed (Figure 1C). Solid organoids appeared as cellular clusters without a central cavity. Hollow organoids displayed a luminal structure. Organoids with a mix of solid and hollow features or diverse morphologies were classified as mixed group. Among 36 successful cases, 50% were solid, 47% were mixed, and only 3% were hollow. Representative bright field images and subtypes of PDOs are detailed in Figure S1 and Table S2 (Supporting Information).

To compare the histological features between PDOs and parental tumors, we performed IHC staining and IF staining. We evaluated the expression of basal markers (cytokeratin (Ck) 5, p63), luminal markers (Ck8, Ck20), a tumor initiating marker (CD44), a proliferating marker (Ki67), and urothelial carcinoma markers (GATA3, uroplakin II (UPKII) (Figure 1D; Figure S2, Supporting Information). H&E staining showed good concordance in histological characteristics between PDOs and parental tumors. The positive results of GATA3 and UPKII staining confirmed that our PDOs originated from urothelial carcinoma. The expression of CD44 and Ki67 further validated the strong stemness and cell viability of our PDOs. In addition, the protein expression level of different markers were mostly consistent between PDOs and parental tumors (**Table** **1**). However, some differences were noted. For example, the parental tumor of BCORG45 exhibited positive expression of basal markers CK5 and p63, while the PDOs were only positive for p63, suggesting that some cell groups may have been lost during the primary culture process. For the luminal or basal subtype, the phenotype of parental tumors characterized by the predominant expression of either luminal or basal markers was mostly observed in PDOs (Table 1). While most PDOs only expressed markers for one subtype, some cases still expressed both basal and luminal markers, indicating the existence of intra‐tumoral heterogeneity.

**Table 1:** Summary of marker expression for indicated markers on different organoid lines.

Line	Marker expression in tissue	Marker expression in organoid	Phenotypic similarity
BCORG1	luminal	luminal	Yes
BCORG2	basal	basal	Yes
BCORG3	mixed	mixed	Yes
BCORG8	basal	basal	Yes
BCORG9	mixed	mixed	Yes
BCORG14	basal	basal	Yes
BCORG16	basal	mixed	Yes (partial)
BCORG23	basal	basal	Yes
BCORG38	luminal	luminal	Yes
BCORG43	basal	mixed	Yes (partial)
BCORG45	mixed	mixed	Yes

### 2.3 PDOs Have Successfully Recapitulated Genomic and Transcriptional Features of Parental Tumors

**Figure 2:**
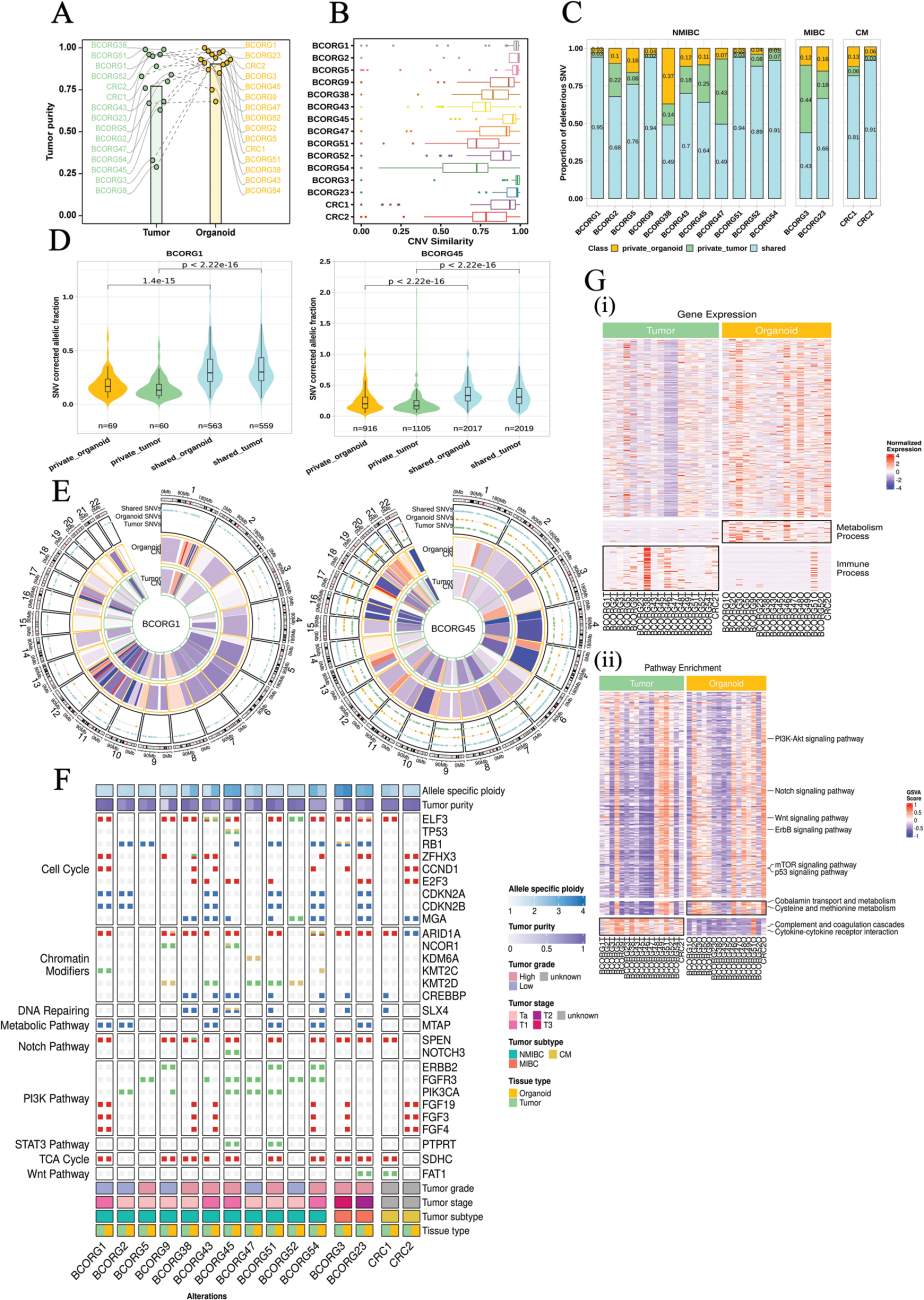
Genomic analysis and transcriptome analysis between 15 PDOs and parental tumors. A) Comparison of tumor purity between PDOs and parental tumors. B) Allele specific CNV similarity of matched tumors and PDOs. C) Proportion of shared and private deleterious SNVs between PDOs and parental tumors. CM: colon cancer metastasis. D) Corrected allelic fraction of all shared and private SNVs in PDOs and parental tumors (BCORG1 left, BCORG45 right). The count of SNVs within each classification is specified. Box plots indicate median, interquartile range, and percentiles. Two‐sided Wilcoxon test. E) Copy‐number and point mutations profiles. Comparison between PDOs and parental tumors for two representative samples (BCORG1 left, BCORG45 right). F) Genomic profiling confirms that PDOs have successfully recapitulate common mutational alterations from parental tumors. G) Analysis of (i) different expression genes and (ii) pathway enrichment between organoids and parental tumors. The colored bar illustrates log2‐transformed values. Different expression genes: p < 0.05, |log2 (fold change)| > 1.

To compare the genomic features between PDOs and matched parental tumors, we analyzed the tumor purity of PDOs and parental tumors. Most PDOs (12 out of 15) had higher purity than their corresponding parental tumors (**Figure** **2**A). Using the methods reported by Martina et al,^[^ ^7c^ ^]^ we calculated a copy‐number‐based similarity score between PDOs and paired parental tumors (Figure 2B). All cases showed good similarity between matched PDOs and parental tumors (copy number variant similarity >50%). To further explore the genomic consistency between PDOs and parental tumors, we analysed the proportion of deleterious single nucleotide variants (SNVs) in each sample. On average, shared mutation accounted for 74.7% (±18.0%), whereas PDOs‐speicfic and parental tumor‐specific mutations account for 10.3% (±8.8%) and 14.9% (±13.7%), respectively (Figure 2C). By comparing the allelic fraction of shared and private SNVs, we found that shared SNVs had a higher value than specific SNVs in each sample (Figure 2D; Figure S3, Supporting Information), indicating that most tumor cells shared mutations. Copy‐number and point mutations profiles of each case showed overall concordance between paired samples (Figure 2E; Figure S4, Supporting Information).

Subsequently, a comprehensive summary of somatic mutations in the PDOs and parental tumors showed that PDOs successfully recapitulated prevalent genomic alterations observed in the matched parental tumors (Figure 2F). Among genes crucial for cell cycle regulation,^[^ ^9^ ^]^ ELF3 had the highest mutation rate (11 out of 15 cases), and a previous paper also reported that ELF3 is one of the most frequently mutated genes in bladder cancer.^[^ ^10^ ^]^ The co‐deletion of CDKN2A/B occurred in 40% of cases, which is associated with tumor recurrence in certain cancer types.^[^ ^11^ ^]^ In addition, some commonly mutated genes in bladder cancer, such as RB1, ZFHX3, CCND1, E2F3, and MGA, also exhibited notable mutation frequencies in our sample. Among chromatin modifiers, we found that some genomic alterations occurred in both NMIBC and MIBC samples, such as ARID1A and KMT2D. Specifically, ARID1A amplifications occurred in 67% of cases, making it one of the most common mutations in patients with bladder cancer.^[^ ^12^ ^]^ Our samples also exhibited some common genomic mutations reported in bladder cancer,^[^ ^13^ ^]^ such as SLX4, MTAP, SPEN, and SDHC. These genes play essential roles in distinct tumor‐related pathways. According to previous reports, some mutations of PI3K‐AKT pathway genes are key drivers of dysregulated growth in NMIBC,^[^ ^13^ ^]^ especially PIK3CA and FGFR3 genes. In our cohort, missense mutations of PIK3CA and FGFR3 genes were identified in 45% and 54% of NMIBC cases, respectively. Conversely, no point mutations of PIK3CA and FGFR3 genes were detected in MIBC cases.

We compared gene expression profiles between parental tumors and PDOs to assess the maintenance of gene expression during culture process, especially oncogenic genes. Quantitative analysis showed a high degree of concordance between matched PDOs and parental tumors in terms of gene expression (Figure S5, Supporting Information). Furthermore, the results revealed that most gene expression profiles were conserved in PDOs, except for immune and metabolic pathways (Figure 2G). Additionally, pathway enrichment analysis revealed significant upregulation of certain oncogenic pathways, including PI3K/AKT, Notch, p53, and mTOR signaling pathways in PDOs.^[^ ^14^ ^]^ These findings suggest that our PDOs possess pronounced tumor characteristics, indicating their potential to be valuable models for bladder cancer research (Figure 2G).

### 2.4 BC‐PDOs Show Different Sensitivity to Chemotherapeutics and Targeted Drugs

**Figure 3:**
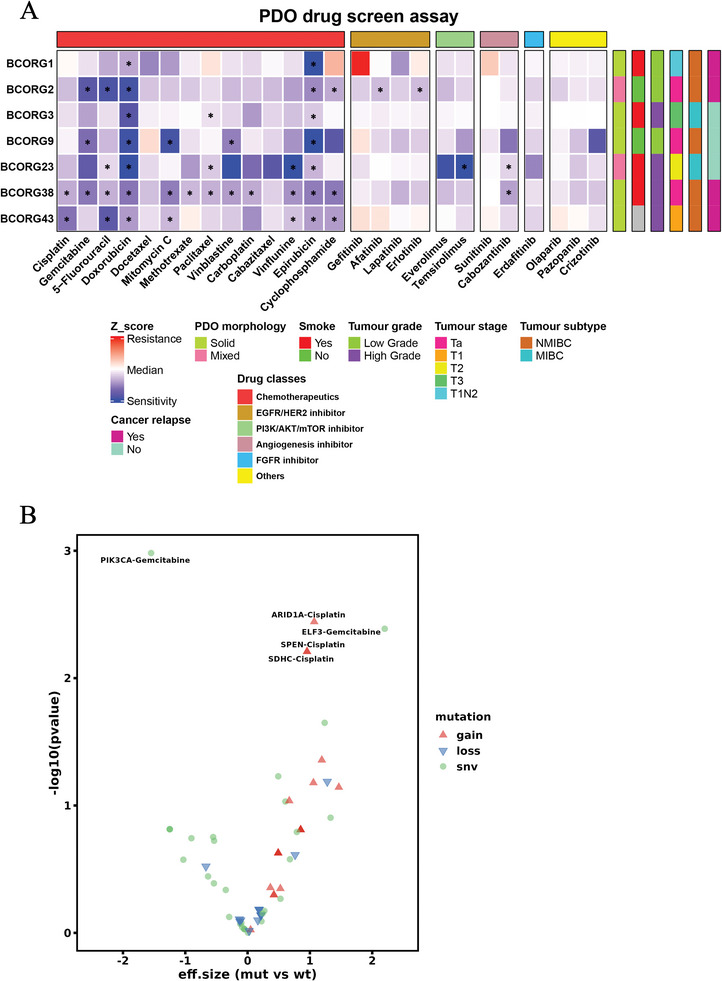
Drug response to chemotherapy or targeted drugs observed in ex vivo bladder cancer organoids. A) The Heatmap shows the results of PDOs drug screening (*n* = 7). Mean z‐scores, normalized to the vehicle values, were obtained from cell viability assays after 72‐h drug exposure for each PDO, are calculated for a single experiment corresponding to each PDOs. Relevant clinical information and PDO morphology are annotated with distinct colors at the bottom of the heatmap. Statistical significance between treatments and vehicle was calculated by one‐way ANOVA test with Dunnet's multiple comparison, a response is indicated by *(z‐score ≤ −1.5, adjusted p‐value < 0.05 and 1‐FC > 0.5). B) Association between genomic mutations and sensitivity/resistance to cisplatin or gemcitabine.

To assess drug response profiles of BC‐PDOs, we conducted drug screening to test their sensitivity to different chemotherapeutic and targeted drugs. We used the maximum blood concentration (Cmax) of each drug as the testing concentration (Table S3, Supporting Information), as it better simulates the in vivo drug effects and allows us to test more drugs.^[^ ^7c^ ^]^ PDOs were considered sensitive when the drug significantly reduced cell viability (z‐score ≤ −1.5, adjusted p‐value < 0.05, and 1‐FC > 0.5). Among the seven tested cases, PDOs showed heterogeneous responses to chemotherapeutic or targeted drugs (**Figure** **3**A). The effects of some drugs were also confirmed by organoid‐formation assay (Figure S6, Supporting Information).

For chemotherapeutic drugs, some drugs showed no significant effect in tested PDOs, such as cabazitaxel and docetaxel (Figure 3A). However, doxorubicin and epirubicin were effective in all PDOs. In addition, drug response statuses observed in PDOs were validated in corresponding follow‐up patients. For instance, one cisplatin‐resistant case (BCORG1) came from a patient who received cisplatin + gemcitabine combination treatment and experienced tumor progression (Figure 5D). Notably, BCORG1 showed limited sensitivity to most chemotherapeutic drugs, except doxorubicin and epirubicin. In contrast, BCORG38 was responsive to most chemotherapeutic drugs, except docetaxel and cabazitaxel. As first‐line treatments, cisplatin and gemcitabine single treatment showed unsatisfactory results, with only 2 out of 7 samples and 3 out of 7 samples showing sensitivity, respectively. Most targeted drugs did not significantly affect the tested PDOs. Despite certain PDOs having mutations in relevant genes, the efficacy of corresponding inhibitors remains poor. Specifically, FGFR inhibitor erdafitinib was ineffective, as also reported by Martina et al.^[^ ^7c^ ^]^ However, BCORG2, which did not respond well to most chemotherapeutic drugs, was significantly sensitive to EGFR/HER2 inhibitors. We analyzed the drug response and genomic profiles to explore the association between genomic mutations and drug sensitivity(Figure 3B). According to our analysis, PDOs with deleterious mutations in PIK3CA gene would be more sensitive to gemcitabine than other PDOs. Conversely, PDOs with deleterious mutations in ELF3 gene would be more resistant to gemcitabine treatment. PDOs showing copy‐gain in ARID1A, SDHC, or SPEN genes would have higher resistance to cisplatin. These findings have illuminated the potential mechanisms involved in developing drug resistance in bladder cancer, thus paving the way for discovering new therapeutic methods.

### 2.5 PDOs and PDO xenograft (PDOX) Are Useful Models to Predict the Drug Response of Corresponding Patients

**Figure 4:**
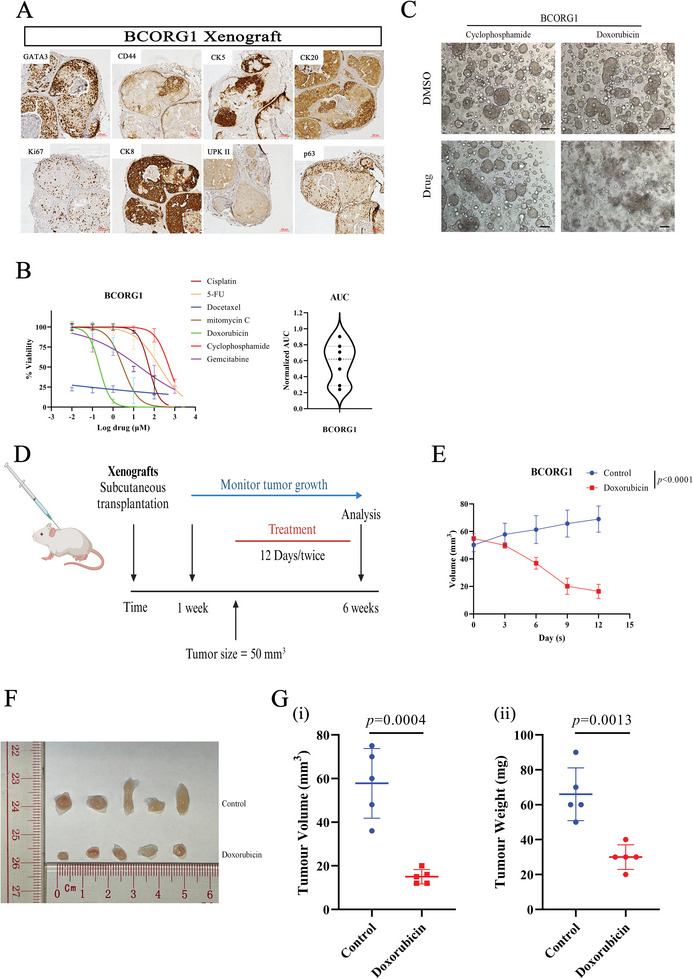
Tumorigenesis in organoid‐derived xenografts and clinically relevant responses to chemotherapy of PDOs in vivo. A) H&E and IHC staining of representative xenograft generated from organoids. Scale bar: 50µm. B) Sensitivity profile of BCORG1 to different chemotherapeutic drugs. Raw data were obtained from cell viability assays after a 72‐h exposure of PDOs to drugs (*n* = 7). The violin plot showed the AUC of each case. C) Effects on the viability of treatment with cyclophosphamide (resistant) or doxorubicin (sensitive) using an organoid formation assay. Scale bar: 100 mm. D) Schematic of in vivo experiments. PDOs were subcutaneously transplanted into NSG mice. These mice would be treated with specific drugs or vehicles every 6 days once the tumor size reached 50–100 mm^3^. E) Tumor growth curves in NSG mice. Mice were treated with doxorubicin, or vehicle every 6 days for 12 days (*n* = 5). Results are shown as the tumor volume (mean ± SD). F) Representative image of BCORG1 xenografts after 12 days of treatment (doxorubicin or vehicle, *n* = 5). G) Comparison of (i) tumor volume and (ii) weight between control and doxorubicin‐treated groups. The statistical differences between groups were analyzed using unpaired two‐tailed t‐test. IC50 and AUC values were analyzed by GraphPad Prism 9 software with nonlinear regression (curve fit) and the equation log(inhibitor) versus normalized response. Error bars represent ± SEM.

To assess the tumorigenic ability of our PDOs, we used them to form ectopic xenografts in vivo. Five cases successfully formed tumor (Table S2, Supporting Information). We conducted IHC staining analysis with a panel of markers (GATA3, Ck5, Ck8, Ck20, CD44, p63, UPKII, Ki67) on the xenografts to compare their characteristics with their corresponding PDOs and found that the immnunoexpression patterns of all PDOXs kept a high concordance with their corresponding PDOs (**Figure** **4**A; Figure S7, Supporting Information). However, we also found that the BCORG1 xenograft was positive to Ck5, which differed from its corresponding PDOs.

Then, we used the PDOX mice model to validate the in vitro drug sensitivity results in PDOs. We chose doxorubicin as the testing drug based on the in vitro drug sensitivity results (Figure 4B,C). BCORG1 cells were injected subcutaneously into NSG mice. Once the tumors reached an appropriate size, the mice were injected intraperitoneally with either drugs or a control vehicle (Figure 4D). As anticipated, there was a significant difference in tumor growth was noted between the treatment and control groups in BCORG 1 (Figure 4E,F). tumor burdens, reflected by tumor weight and volume, were significantly reduced in the doxorubicin treatment group (Figure 4G). These results indicated that the drug screening findings in PDOs were replicated in PDOX mice.

**Figure 5:**
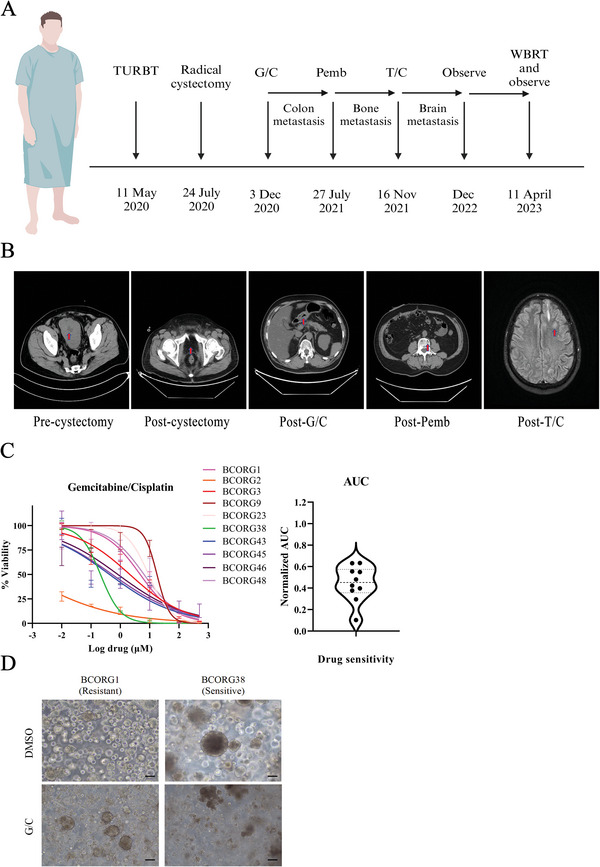
PDOs have the potential to predict the drug sensitivity of corresponding patients. A) Timeline schematic of patient BCORG1 treatment stages.G/C = gemcitabine/cisplatin;Pemb = Pembrolizumab;T/C = docetaxel/cyclophosphamide; WBRT = whole brain radiotherapy. B) CT scan at different stages of patient BCORG1 treatment. Tumor lesions are marked with red arrows. C) Summary of drug sensitivity for 10 PDOs to combined therapy (gemcitabine + cisplatin, G/C). Results are displayed as dose‐response curves. The violin plot showed the AUC of each case (*n* = 10). D) Effects on the viability of treatment with G/C using an organoid formation assay. Scale bar: 100 µm. IC50 and AUC values were analysed by GraphPad Prism 9 software with nonlinear regression (curve fit) and the equation log(inhibitor) versus normalized response. Error bars represent ± SEM.

In addition, we compared the drug screening results with actual clinical outcomes. According to our findings, PDOs showed heterogeneous sensitivity to the combined therapy (gemcitabine + cisplatin, G/C), which is also observed in bladder cancer patients. Among these patients, patient BCORG1 experienced adjuvant chemotherapy (G/C) after surgery and developed colon metastasis, indicating clinical resistance to G/C treatment (**Figure** **5**A,B). This outcome was consistent with the drug response in corresponding PDOs (Figure 5C,D). These results further demonstrate that PDOs could be a valuable tool for predicting drug response in BC patients.

### 2.6 Gene expression Profiles of BC PDOs in Relationship with Cisplatin or Gemcitabine Response

**Figure 6:**
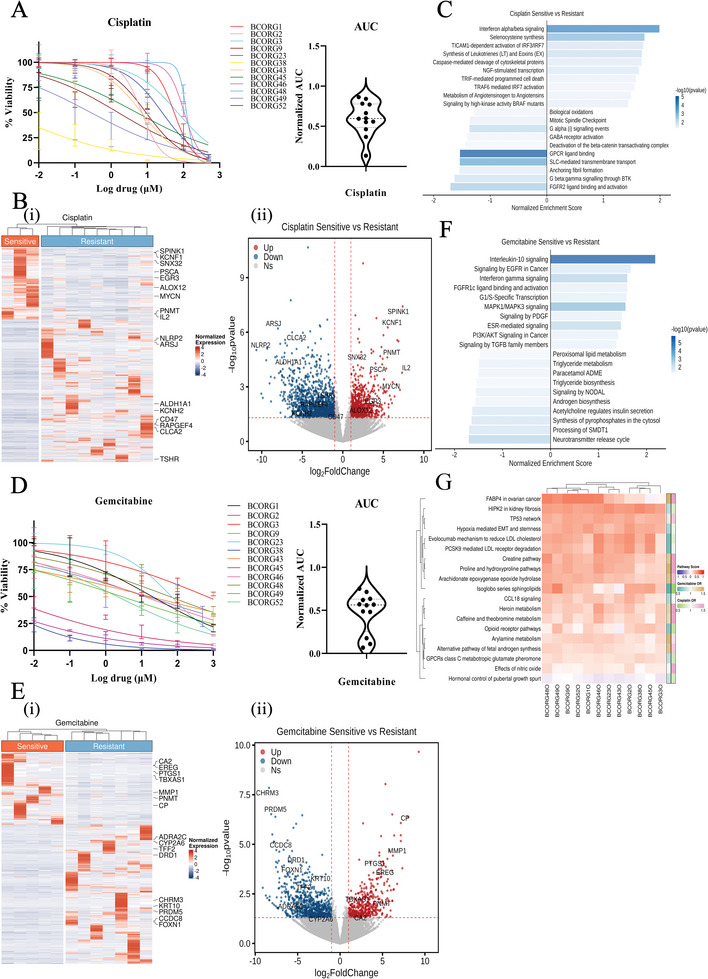
Transcriptional profiling of different response cohorts to cisplatin or gemcitabine treatment. A) Ex vivo drug sensitivity of PDOs to cisplatin shown as dose‐response curves. The violin plot showed the AUC of each case. B) (i) Heatmap and (ii) volcano analysis showed the most differentially expressed genes in the cisplatin sensitive group (*n* = 3) compared to cisplatin‐resistant group (*n* = 9). Sensitive group (AUC < 0.5); Resistant group (AUC ≥ 0.5). Differentially expressed genes: pvalue < 0.05, log2FoldChange > 1. C) Gene set enrichment enrichment analysis of cisplatin‐sensitive group (AUC < 0.5) compared to cisplatin‐resistant group (AUC ≥ 0.5). The colored bar represents the ‐log10(p‐value). D) Ex vivo drug sensitivity of PDOs to gemcitabine in the form of dose‐response curves. The violin plot showed the AUC of each case. E) (i) Heatmap and (ii) volcano plot analysis showed the most differentially expressed genes in the gemcitabine sensitive group (*n* = 5) compared to gemcitabine‐resistant group (*n* = 7). Sensitive group (AUC < 0.5); Resistant group (AUC ≥ 0.5). Differentially expressed genes: p‐value < 0.05, log2FoldChange > 1. F) Gene set enrichment analysis of gemcitabine sensitive group (AUC < 0.5) compared to gemcitabine‐resistant group (AUC ≥ 0.5). The colored bar represents the ‐log10(p‐value). G) Differentially activated pathways between sensitive and resistant groups for cisplatin and gemcitabine. The pathway score was the ssGSEA score which was measured from Wikipathways. The odds ratio (OR) was the beta value from the linear model of (pathway score ∼ drug AUC). IC50 and AUC values were analyzed by GraphPad Prism 9 software with nonlinear regression (curve fit) and the equation log(inhibitor) versus normalized response. Error bars represent ± SEM.

To investigate the underlying mechanism of drug resistance to cisplatin and gemcitabine, we did RNA sequencing of 12 PDOs and analyzed their gene expression profiles. Based on the dose‐response curves, the 12 PDOs were stratified into sensitive or resistant groups according to their sensitivity to cisplatin (sensitive: normalized AUC <0.5, resistance: normalized AUC ≥ 0.5) (**Figure** **6**A). By comparing the cisplatin‐sensitive group (*n* = 3) with cisplatin‐resistant group (*n* = 9), we found that genes associated with cell apoptosis or cisplatin sensitivity, such as SPINK1, KCNF1, PSCA, ALOX12, MYCN, were significantly up‐regulated in the sensitive group (Figure 6B). Conversely, genes associated with cisplatin resistance, such as NLRP2, ALDH1A1, CD47, and CLCA2, were down‐regulated in the sensitive group. Pathway enrichment analysis revealed that certain cell apoptosis‐related pathways, such as TRIF‐mediated programmed cell death and caspase‐mediated cleavage of cytoskeletal proteins were activated in cisplatin‐sensitive group (Figure 6C).

We also classified 12 PDOs into two groups based on their sensitivity to gemcitabine (sensitive: normalized AUC < 0.5, resistance: normalized AUC ≥ 0.5) (Figure 6D). By comparing the gemcitabine‐sensitive group (*n* = 5) with the resistant group (*n* = 7), we found that genes associated with cell apoptosis or gemcitabine sensitivity, such as CA2, EREG, PTGS1, TBXAS1, MMP1, were significantly upregulated in the sensitive group (Figure 6E). These genes were predominantly enriched in well‐known tumor‐related pathways, such as the EGFR signaling pathway and the PI3K/AKT signaling pathway (Figure 6F). Conversely, genes associated with chemotherapy resistance, such as ADRA2C, and TFF2, were significantly down‐regulated in the sensitive group (Figure 6E). Moreover, WikiPathways analysis indicated a strong connection between TP53 network and cisplatin resistance, the FABP4‐related pathway and gemcitabine resistance (Figure 6G).

### 2.7 BC‐PDOs Could be a Useful Platform to Investigate Tumor Microenvironment

**Figure 7:**
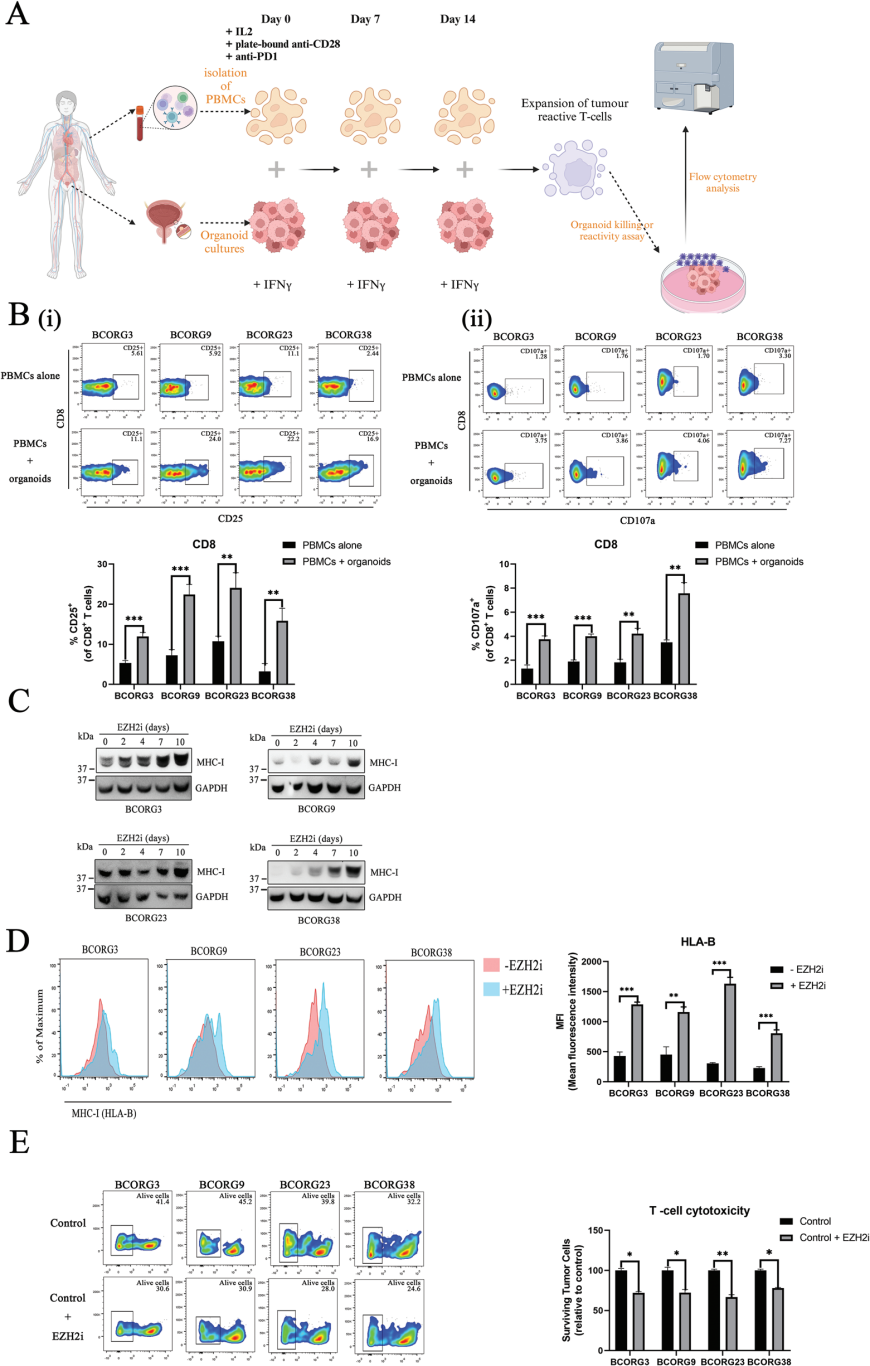
Exploring interactions between tumor‐reactive T cells and tumor cells using the organoid model. A) Experimental workflow of generating autologous tumor‐reactive T cells. PDOs were used to stimulate autologous PBMCs. After two weeks of co‐culture, we successfully isolated the tumor‐reactive T cells from the mixture. (Created in BioRender. Ho, V. (2024) https://BioRender.com/u12w586). B) Representative flow cytometry plots gated on CD8+ T cells tested for (i) activity and (ii) reactivity against autologous organoids after 2 weeks of co‐culture with autologous PDOs. C,D) Cell surface expression level of MHC‐I in EZH2i‐treated organoid cells (EPZ‐011989 5 µm) were analyzed by (C) immunoblotting and (D) flow cytometry after 10 days of treatment. E) Organoids: autologous tumor‐reactive T cells co‐culture assay with EPZ‐011989 inhibition. For pharmacologic inhibition, PDOs were treated with EPZ‐011989(5 µm) or DMSO for 10 days and mixed with autologous tumor‐reactive T cells at an O: T ration of 1: 5. After 24 h of co‐culture, surviving tumor cells were counted by flow cytometry. The statistical differences between groups were analyzed using unpaired two‐tailed t‐test. Error bars represent ± SEM. *p < 0.05; **p < 0.01; ***p < 0.001.

Tumor‐reactive T cells play an important role in tumor microenvironment. To generate tumor‐reactive T cells, we selected four PDOs with autologous PBMCs for co‐culture following the protocol by Krijn K et al.^[^ ^15^ ^]^ (**Figure** **7**A). Before co‐culture, the organoids were treated with 200 ng mL^−1^ IFN‐γ overnight. The treated PDOs were then used to stimulate autologous PBMCs. After two weeks of co‐culture, we isolated the T cells from the mixture using a human T cell isolation kit. Flow cytometry results showed that the activity (CD25+) of isolated T cells was greatly enhanced, and their reactivity (CD107a+) against autologous PDOs was also increased (Figure 7B), confirming we have successfully generated tumor‐reactive T cells using that PDOs co‐culture platform.

To validate the utility of our PDOs co‐culture model, we employed it to investigate the role of EZH2 in the microenvironment of bladder cancer. In the tumor microenvironment, the ability of CD8^+^ T cells to recognize and kill tumor cells was influenced by the interaction between the MHC‐I complex and the T cell receptors (TCRs). According to previous publications, pharmacological inhibition of EZH2 could influence the cell surface expression of MHC‐I (HLA‐A/B/C).^[^ ^16^ ^]^ However, existing studies have only utilized murine‐derived bladder tumors to investigate the relationship between EZH2 and MHC‐II.^[^ ^17^ ^]^ To better reflect the relationship between EZH2 and MHC‐I in bladder cancer, we used PDOs co‐culture model to explore the role of EZH2 in bladder cancer. Based on the growing curve, we selected suitable working concentration which would not directly influence the viability of PDOs (Figure S8, Supporting Information). After that, the results of immunoblotting, flow cytometry, and immunofluorescence confirmed that pharmacological inhibition of EZH2 restored cell surface MHC‐I (HLA‐B or HLA‐A/B/C) in PDOs (Figure 7C,D; Figure S9, Supporting Information), indicating the occurrence of enhanced antigen presentation on the EZH2i‐treated PDOs. Furthermore, we co‐cultured treated PDOs with autologous tumor‐reactive T cells and tested the T‐cell cytotoxicity by flow cytometry, organoid formation assay, and ELISA assay. The results showed that pre‐treatment of EZH2i significantly enhanced the cytotoxicity of tumor‐reactive T cells (Figure 7E; Figure S10, Supporting Information). In addition, the images under fluorescent field or bright field confirmed that pre‐treated EZH2 inhibition help reactive T cells to reduce organoid size and broken organoid structure (Figures S11 and S12, Supporting Information). These results proved that PDOs serve as a valuable model for investigating the interactions among various cells within the tumor microenvironment, offering insights that can aid in the discovering of novel treatment methods.

## 3 Discussion

The treatment methods are different across various clinical stages of bladder cancer.^[^ ^18^ ^]^ However, the oncological outcomes for both NMIBC and MIBC remain unsatisfactory, with a high recurrence rate and various complications.^[^ ^19^ ^]^ In recent years, targeted therapy and immunotherapy, such as erdafitinib and pembrolizumab, have been incorporated into clinical practice. Despite this, many patients do not benefit from these treatment methods, even with relevant genomic mutations.^[^ ^20^ ^]^ These years, researchers have used genomic approaches to explore the inter‐patient heterogeneity of bladder cancer. Some studies have revealed the genomic difference between NMIBC and MIBC and categorized genomic subtypes of bladder cancer.^[^ ^21^ ^]^ However, these findings need to explain the poor treatment outcome fully. To understand the biological function of common somatic mutations, and develop precise and personalized treatment methods, we must establish a pre‐clinical model that includes the known population‐level subtypes of bladder cancer.

In this study, we successfully established a PDOs biobank encompassing a broad spectrum of bladder cancer subtypes. We explored the utility of PDOs in personalized treatment and immunotherapy for bladder cancer patients. Notably, 64% of cases formed organoid structure during the culture process, and most of them were derived from the NMIBC group. We demonstrated that PDOs can recapitulate the morphological and histological features of parental tumors, and our markers analysis also revealed intra‐tumoral heterogeneity. We have also compared the genomic profiles between matched PDOs and parental tumors. We found that the mutational profiles of organoids were highly consistent with not only the parental tumors, but also with the overall molecular signature of human bladder cancer. In addition, our transcriptional analysis indicated that most gene expression profiles were conserved in PDOs. However, WES and RNA‐seq found some dissimilar features between PDOs and parental tumors, potentially due to specific tumor subclonal events, limitations inherent in the culturing process, or technical sequencing constraints.^[^ ^7^, ^22^ ^]^ Overall, the ability of PDOs to mimic parental tumors validates their potential as a robust model for cancer research.

To assess the drug response profiles of BC‐PDOs, we developed a drug screening system including a range of chemotherapeutic and targeted drugs. We found significant variability in drug responses across PDOs, highlighting the intertumor heterogeneity of bladder cancer. Consistent with earlier findings,^[^ ^7c^ ^]^ targeted drugs did not show substantial antitumor effects, even for cases with relevant genomic mutations. Conversely, some chemotherapeutic drugs performed better with broader tumor suppression capability. Then, we used NSG mice to affirm PDOs’ tumorigenic ability and verify in vivo drug sensitivities, confirming the concordance between in vivo and in vitro drug response results. Furthermore, our assessments of drug sensitivity were reflected by the actual clinical outcomes, demonstrating the potential of PDOs in evaluating and predicting tumor responses to different drugs.

Moreover, we integrated transcriptional profiles of PDOs with their drug response data to investigate the potential mechanisms behind varying responses to chemotherapy. This analysis revealed some upregulated genes linked to cellular apoptosis or drug transport in the cisplatin‐sensitive group, including SPINK1 and MYCN.^[^ ^23^ ^]^ Pathways associated with cell apoptosis were activated in this group, including TRIF‐mediated programmed cell death.^[^ ^24^ ^]^ Furthermore, WikiPathways analysis also highlighted a robust correlation between the TP53 pathway and cisplatin response. These findings elucidate the underlying mechanisms of drug resistance, guiding our subsequent research into tumor resistance.

To explore the applicability of PDOs in immunotherapy, we established a PDO‐PBMC co‐culture system and generated autologous tumor‐reactive T cells.^[^ ^7a^ ^]^ To test the practicality of our PDOs co‐culture model, we used it to investigate the influence of EZH2 inhibition, and we demonstrated that it could support autologous tumor‐reactive T cells in killing tumor cells. For this co‐culture system, it may have significant utility in two primary areas.^[^ ^7a^ ^]^ First, it will help researchers to investigate the mechanisms that govern the sensitivity and resistance of bladder cancer to immunotherapy, just like what we have shown. Second, it provides the possibility to achieve patient‐specific immunotherapy. The capacity to derive BC‐PDOs from even the smallest tumor samples, such as those obtained through needle biopsies, coupled with the ability to proliferate circulating tumor‐reactive T cells from peripheral blood, offers a minimally invasive approach to assess the immunotherapy responsiveness of tumors in individual patients at different time points of treatment.

In recent years, some studies have also reported the establishment of PDOs for bladder cancer.^[^ ^7^ ^]^ However, our study reported the world's first PDOs biobank for bladder cancer on an Asian population, and it is the most comprehensive to date and the first to investigate potential mechanisms of cisplatin and gemcitabine response using BC PDOs. According to previous studies, there exists several differences between Asian and Caucasian patient cohorts. Notably, both African American and Caucasian groups have been reported to exhibit lower survival probabilities compared to their Asian counterparts.^[^ ^5a^ ^]^ Furthermore, considering the variations in environmental factors and clinical practice among different regions, the pursuit of truly personalized medicine necessitates the development of PDO biobanks that reflect the genetic and clinical profiles of local patient populations. Several studies have also reported genomic difference between Asian and Caucasian patients.^[^ ^5b,c^ ^]^ For instance, the well‐known driver gene TP53 shows a lower mutation rate in the Asian group, which is also found in our PDOs biobank. Furthermore, our biobank showed a higher success rate in the NMIBC group.^[^ ^7c^ ^]^ As we known, the high recurrence is a significant challenge in bladder cancer, with many patients experiencing frequent relapses after TURBT, ultimately leading to the need for radical cystectomy. The characteristics of our PDO biobank offers hope for rapid postoperative drug screening and genetic testing, which may pave the way for personalized and bladder‐preserving treatment. It may have chance to reduce recurrence rate and enhance the quality of life for patients in the future. Additionally, we are the first to use PDOs to explore the tumor microenvironment of bladder cancer. Our PDOs have some common characteristics with PDOs from other ethnicities, such as the observation that targeted therapies are less effective than traditional chemotherapeutic drugs. At the genomic level, both sets of PDOs harbor common mutations associated with bladder cancer, such as PIK3CA, FGFR3, and RB1. This further demonstrates the strengths of the PDO model. Above all, our study indicates that PDOs could be a valuable tool for region‐specific research, clinical decision support, and improving patient prognoses. PDOs can contribute significantly to preclinical studies, particularly in disease modeling and therapeutic compound identification. Compared to previous studies, we utilized the drug‐response curve and AUC to explore the reasons for different sensitivity to cisplatin or gemcitabine among various PDOs. However, due to the limited number of tested PDOs, further mechanistic studies and validation with a larger cohort of PDOs are necessary. Based on our analysis, we observed a higher success rate in PDO establishment among patients who received TURBT. Further investigation is warranted to explain the underlying mechanisms responsible for this observation. Furthermore, a comprehensive prospective study involving a large cohort of bladder cancer PDOs need to be considered to assess their clinical relevance and inform their potential integration into personalized cancer care strategies. Moreover, as the first PDOs biobank for bladder cancer on an Asian population, our analysis of the difference between Asian and Caucasian populations’ PDOs is still insufficient. Future research should further investigate the disparities among PDOs derived from different populations.

A pressing challenge in bladder cancer management is the development of precise methods to predict treatment efficacy for individual patient. Our team have established the world's first PDOs biobank for bladder cancer based on an Asian population, which paves the way for subsequent research on bladder cancer treatments tailored to Asian populations. This biobank leverages the potential of PDOs to model patient‐specific tumor biology and therapeutic responses, offering a valuable resource for personalized medicine and precision oncology in the context of bladder cancer. For future studies, we need to establish a larger cohort of PDOs to refine and substantiate our findings. This expanded collection will enable more comprehensive analysis and bolster the reliability of our results.

## 4 Experimental Section

### Sample Collection

The study was approved by the institutional ethical committee (Joint Chinese University of Hong Kong‐New Territories East Cluster Clinical Research Ethics Committee, No. 2016.396). Informed consent was taken from the patients. Parental tumor tissue was obtained through TURBT or radical cystectomy procedures. Part of the parental tumor tissues were saved to establish PDOs, and a cold basal medium supplemented with 10% fetal bovine serum (FBS, Gibco) and 10 µm ROCK inhibitor was used as the transport medium. The remaining tissue samples were examined by a dedicated uropathologsts.

### Tissue Dissociation and Organoid Culture

The procedure of organoid primary culture followed the protocol described by Jasper et al.^[^ ^22^ ^]^ Some modifications were made to improve the overall viability of the tumor cells. After washing, the tissues were finely minced and digested at 37 °C for 30–60 min. Then the digestion process was stopped using Adv DMEM/F12 containing 10% FBS. Then, the tissue mixture was further digested by TrypLE Express supplemented with ROCK inhibitor at 37 °C for 5 min. Afterward, the mixture was passed through a 70 µm cell strainer (Corning). After that, the cell pellet was mixed with the Matrigel (Corning) and seeded into the well. Once the Matrigel solidified, organoid culture medium was added. The culture medium was changed twice weekly, and organoids were passaged every 10 days.

### Immunohistochemistry Staining, Immunofluorescence Staining, and Immunoblotting

Both organoids and tissues were fixed in 4% paraformaldehyde for at least 1 h, dehydrated, and paraffin‐embedded using standard histology procedures. The sections were stained with Hematoxylin and Eosin (H&E). Immunohistochemistry (IHC) staining and immunofluorescence (IF) staining were performed with primary antibodies, including Anti‐CK5 (1:250, Abcam), Anti‐CK20 (1:250, Abcam), Anti‐Ki67 (1:250, Abcam), Anti‐GATA3 (1:250, Abcam), Anti‐UPKII (1:250, Abcam), Anti‐CK8 (1:250, Abcam), Anti‐p63 (1:250, Abcam), Anti‐CD44 (1:250, Abcam). For immunoblotting, total protein was extracted from organoids using RIPA buffer (supplemented with phosphatase inhibitor and protease inhibitor). The primary antibodies include Anti‐GAPDH (1:2000, CST), Anti‐HLA‐I (1:1000, Abcam).

### Drug Screening and Cell Viability Assay

Organoids at passages P3‐P10 from available BC PDOs were used for drug screening. They were dissociated into single cells or small clusters using TrypLE Express, filtered through a 70µm cell strainer to eliminate large organoids. Cells were then seeded in at least 3 replicates for each drug condition, with positive (Staurosporine) and negative (DMSO or H_2_O) controls in a ULA 384‐well plate (Corning) in organoid culture medium containing 2% Matrigel (10 000 orgnaoids mL^−1^). Each well would be seeded 1500–3000 organoids. After 48 h of culture, each drug was diluted in organoid culture medium and added to each well at a final concentration of 1xCmax reported in previous publications. The final concentration of DMSO or H_2_O solvent in the treatment condition was 0.5%. After 72 h of drug treatment, cell viability was measured using the CellTiter‐Glo 3D assay (Promega) according to the manufacture's instructions. Raw values were individually standardized for each specimen using the equation: (X_s_ – X_v_)/SD_v_, where X_s_ represents the technical replicates for each drug; X_v_ denotes the mean of technical replicates from corresponding vehicles and SD_v_ is the standard deviation of the technical replicates from matching vehicles. The Z‐factor was used as a statistical indicator of drug screening assay quality and calculated as 1‐ [3x(SD of sample + SD of control)/(Mean of sample – Mean of control)]. The Z‐scores for cisplatin, carboplatin, and 5‐FU were calculated using H_2_O as the negative control, while all other drugs used DMSO as the negative control. Additionally, raw values were used to calculate fold‐changes relative to the mean of raw values from the vehicles.

To evaluate chemotherapy drug responses, organoids were digested and seeded in ULA 384‐well plate (Corning) at a density of 2500 cells per well in 2% Matrigel/culture medium. After 48 h, fresh organoid culture medium with varying drug concentrations was added. The maximal vehicle concentration used was <5%. After 72 h, cell viability was assessed using the CellTiter‐Glo 3D assay (Promega). IC50 and AUC values were analyzed by GraphPad Prism 9 software with nonlinear regression (curve fit) and the equation log(inhibitor) versus normalized response.^[^ ^25^ ^]^ Each concentration was tested in at least 3 replicates.

### Whole‐Exome Sequencing and Analysis

DNA was extracted from the buffy coat, tumor tissue, and organoid samples using either the QIAamp DNA/RNA mini kit (Qiagen) or QIAamp DNA blood mini kit (Qiagen) as per the manufacturer's instructions. The extracted DNA was then quantified using Qubit dsDNA HS Assay Kits (Invitrogen) and a Qubit 4 Fluorometer. The Whole‐exome sequencing libraries were prepared using the SureSelect Human All Exome V6 (58M) (Agilent Technologies) and VAHTS Universal Pro DNA Library Prep Kit for illumina V2 (Vazyme). The libraries were sequenced on an Illumina platform using the NovaSeq 6000 instrument at a read length of 2 × 150 bases.

### DNA Sequencing Analysis

The raw DNA sequencing reads were quality checked by FastQC (v0.11.4) and processed to filter the low‐quality reads using FastP (v0.23.4).^[^ ^26^ ^]^ Then, clean reads were mapped against the human genome assembly (GRCh37/hg19) by BWA mem algorithm (v0.7.17)^[^ ^27^ ^]^ with default settings. The bam files were sorted by samtools (v1.19),^[^ ^28^ ^]^ marked the duplicates by picard tools (v1.14) and did the indel realignment and base recalibration by the Genome Analysis Toolkits (GATK v3.4).^[^ ^29^ ^]^ Somatic SNVs and indels were called by Mutect (v1.1.7),^[^ ^30^ ^]^ Platypus (v1.0),^[^ ^31^ ^]^ Strelka (v1.0)^[^ ^32^ ^]^ and Scalpel (v0.5.2).^[^ ^33^ ^]^

The confident mutations were retained by the following criteria: 1) not detected in normal sample panel and dbSNP135;^[^ ^34^ ^]^ 2) variant allele frequency more than 0.05.

Allele specific copy number variations (CNVs) assessment based on segmentation by CNVkit (v0.9.10) and CLONET v2 (v2.2.1). The CNV similarity of PDOs (o) and parental tumors (t) were calculated by:

1. d = ||(cnAt, cnBt) – (cnAo, cnBo)|| ((cnA, cnB) being the major and minor alleles of a sample for each gene;d is the Euclidean distance of PDOs and parental tumors)
2. d’ = (d‐min(d))/(max(d)‐min(d)) (d’ is the normalized distance);
3. s = 1 – d’ (s is the similarity)

The final mutations were annotated by SnpEff,^[^ ^35^ ^]^ SnpSift (v3.6a)^[^ ^36^ ^]^ and VEP (v105).^[^ ^37^ ^]^

Tumor purity of PDOs and parental tumors were measured by the TPES (v1.0.0).^[^ ^38^ ^]^

The association between genomic mutations and drugs activity was tested by random intercept Liner Mixed Model (LMM): z ∼ β_1_x + β_0_m, where z is vector of z‐scores for a drug across all replicates of samples, x is the vector which encoding the mutation status (0 for wild type and 1 for mutated), m is a vector that including the sample info for each replicate. The effect size was obtained from the estimated β_1_ of association result and p‐values was measured by the Likelihood Ratio Test that comparing the above model to the null model. The LMM analysis was implemented by R package lme4 (v1.1.35.5).

### RNA‐Sequencing and Analysis

Total RNA was extracted from fresh tumor tissue, FFPE samples, and organoids using QIAamp DNA/RNA mini kit (Qiagen) or HiPure FFPE Plus kit (Magen) as per the manufacturer's instructions. The extracted RNA was then quantified using Qubit RNA HS Assay Kits (Invitrogen) and a Qubit 4 Fluorometer. The RNA was first subjected to ribosomal RNA depletion using Ribo‐off rRNA depletion kit (Human/Mouse/Rat) (Vazyme), followed by library preparation by VAHTS Universal V8 RNA‐seq Library Prep kit for Illumina (Vayzme). The sequencing was performed using Novaseq 6000 instrument (Illumina) at a read length of 2 × 150 bases.

The raw RNA reads were quality checked by FastQC (v0.11.4) and processed to filter the low‐quality reads using FastP (v0.23.4).^[^ ^27^ ^]^ The remaining reads were aligned to human reference genome GRCh38/hg38 using STAR (v.2.7.11a).^[^ ^39^ ^]^ Gene expression level were quantified using RSEM (v1.3.3).^[^ ^40^ ^]^ The spearman correlation coefficient and R‐squared were used to assess the concordance of PDOs and parental tumors at the gene expression level. Then, the gene counts matrix was input into DEseq2 (v1.40.2)^[^ ^41^ ^]^ for differential expression analysis. The GSVA scores were calculated by the GSVA package (v1.46.0)^[^ ^42^ ^]^ with the method set to “gsva”. Gene set enrichment analysis was implemented in clusterProfiler (v4.6.2).^[^ ^43^ ^]^ The odds ratio (OR) was from beta value of linear model: pathway score ∼ drug AUC, which uses lm() function from the R base package. The pathway score was the ssGSEA score which calculated by GSVA (v1.46.0) with the ssGSEA method and pathways were sourced from the WikiPathways database.^[^ ^44^ ^]^

### PDO Xenograft Models and Chemotherapy in NSG Mice

All mouse experiments were conducted following the guidelines approved by the institutional animal ethics committee (Animal Experimentation Ethics Committee in The Chinese University of Hong Kong, No. 22‐018‐MIS). For PDOX, each 6‐8‐week‐old male NOD.Cg‐Prkdc^scid^ Il2rg^tm1Wjl^/SzJ (NSG) mice were subcutaneously injected with 2 × 10^6^ BC PDOs mixed with 75ul Matrigel and 25ul Adv DMEM/F12. tumor dimensions were measured every third day, and tumor volume was calculated as per previous studies.^[^ ^8^ ^]^ Once the tumor reached 50 mm^3^, mice were randomly assigned to treatment (doxorubicin) or control groups (physiological saline). Doxorubicin (4mg kg^−1^) or saline was administered intraperitoneally every 6 days. After 12 days, mice were sacrificed, and fresh tumors were harvested for subsequent experiments.

### Generation of Tumor‐Reactive T Cells

The patient's blood was obtained from the patients after obtaining informed consent. After blood collection, peripheral blood mononuclear cells (PBMCs) were isolated using Fico‐Paque, following the protocols by Bain B *et* al.^[^ ^45^ ^]^ The isolated PBMCs were mixed with cryogenic medium (10% DMSO + 90% FBS) and transferred into a cryovial. The tubes were placed in Mr. Frosty tube racks and immediately stored in a −80 °C freezer overnight.

To generate the tumor‐reactive T cells, the protocol by Krijin K et al., was followed.^[^ ^15^ ^]^ to establish a co‐culture system. The day before co‐culture, the culture medium for PBMCs was prepared, consisting of RPMI 1640 (GIBCO), 1:100 PSN, and 10% FBS (GIBCO). Cryopreserved PBMCs were defrosted in 37 °C T cells culture medium and treated with 25 U mL^−1^ benzonase (Merck) to prevent cells from clumping. After that, the PBMCs were resuspended in the culture medium with 150 U mL^−1^ interleukin‐2 at 2–3*10^6^ cells mL^−1^, and incubated at 37 °C. Meanwhile, 200 ng mL^−1^ human recombinant interferon‐gamma (IFN‐γ) (Peprotech) was used to stimulate the PDOs, and used 5 mg mL^−1^ anti‐CD28 (clone CD28.2, eBioscience) to coat the 96‐well U‐bottom plates. The next day, stimulated organoids were digested and resuspended in T cell culture medium. PBMCs, prepared the previous day, were collected, counted, and mixed with organoids at a 20: 1 PBMCs‐to‐organoids ratio. After washing the anti‐CD28‐coated U‐bottom plates twice with PBS, the mixture was seeded at a density of 10^5^ PBMCs/well. To enhance organoid stimulation of T cells, the co‐culture system was supplemented with 150 U mL^−1^ IL‐2 and 20 mg mL^−1^ anti‐PD‐1‐blocking antibody (kindly donated by Invitrocue, HK). The culture medium (supplemented with IL‐2 and anti‐PD‐1) was renewed 2–3 times weekly. After 7 days, PBMCs were collected, counted, and co‐cultured with fresh organoids. The entire co‐culture process lasted 14 days, after which the mixture would be collected and washed with PBS. For organoid killing assay, T cells were isolated from co‐culture PBMC using the human T cell isolation kit (EasySep) and cultured in T cells culture medium supplemented with IL‐2.

### Flow Cytometry

To evaluate the expression levels of HLA‐I and PD‐L1 on PDOs, TrypLE Express was used to dissociate organoids following the digestion protocol. Before collecting organoid cells, they were pre‐incubated with specific concentrations of IFN‐γ or an EZH2 inhibitor. After digesting the organoids into single cells, it was washed twice with FACS buffer (PBS + 1% Bovine serum albumin) and stained them with anti‐human HLA‐A, B, C‐PE (eBioscience), anti‐human HLA‐B‐PE (Miltenyi Biotec) or anti‐CD274‐APC (eBioscience) antibodies for 30 min on ice. The mixture was then washed and stained with Fixable Viability Dye to exclude dead cells. Samples were analyzed using a BD LSRFortessa cell analyzer.

To evaluate tumor reactivity, 5 × 10^4^ single organoids were mixed with co‐cultured PBMCs at a 1:2 target‐to‐ effector ratio and seeded in an anti‐CD28‐coated U‐bottom plate with 20 mg mL^−1^ anti‐PD‐L1 and anti‐human CD107a‐PE antibodies (BD). Organoids were pre‐treated with IFN‐γ for 24 h. After co‐culturing for 5 h, cells were collected and washed twice in FACS buffer, and then blocked with an Fc receptor binding inhibitor for 20 min on ice. Cells were then stained with the following antibodies: anti‐CD3‐Pacific blue (Invitrogen), anti‐CD4‐FITC (BD), anti‐CD8‐APC (BD), and anti‐CD25‐Alexa Fluor 700 (Invitrogen). The mixture was subsequently stained with Fixable Viability Dye to exclude dead cells and analyzed using a BD LSRFortessa cell analyzer.

### Organoid Killing Assay

For the organoid killing assay, organoids were pre‐treated with EZH2i EPZ‐011989 (5µm) for 10 days and dissociated into single cells. The organoids and autologous tumor‐reactive T cells were mixed at 1:5 organoids‐to‐ T cells ratio and seeded in an anti‐CD28‐coated U‐bottom plate. After 24 h, cells were collected and stained with anti‐CD3‐Pacific blue (Invitrogen) and Fixable Viability Dye. The surviving tumor cells were counted using BD LSRFortessa cell analyzer. The media of the co‐culture system was collected to test for IFN‐γ levels using the human IFN‐γ ELISA kit (Invitrogen).

### Statistical Analysis

Sample size (n) for each statistical analysis is provided in the relevant figure legends. The pre‐processing and analysis of sequence data were described in the Experimental Section. IC50 and AUC values were analyzed by GraphPad Prism 9 software with nonlinear regression (curve fit) and the equation log(inhibitor) versus normalized response.^[^ ^25^ ^]^ Each concentration was tested in at least 3 replicates. In Figure 3, statistical significance between treatments and vehicle was calculated by one‐way ANOVA test with Dunnet's multiple comparison, a response is indicated by *(z‐score ≤ −1.5, adjusted p‐value < 0.05 and 1‐FC > 0.5). Experiments in Figure 7B–E were performed in triplicates. In this study, data were plotted as mean ± standard deviation, with the student's t‐test and one‐way ANOVA were applied to compare two groups and multiple groups, respectively. Statistical analyses were conducted by GraphPad Prism 9 software. *p < 0.05; **p < 0.01; ***p < 0.001; ns, not significant.

## Conflict of Interest

The authors declare no conflict of interest.

## Author Contributions

Z.H. conducted experiments and wrote the manuscript. L.N. conducted bioinformatic analysis. H.V.W.S., L.K., C.X., W.H., L.H., Z.D., K.L.W., and H.F.C. helped to do primary culture and mice experiments. P.K.‐F.C., I.C.‐H.K., C.H.‐M.W., D.K.‐W.L., and S.K.‐K.Y. prepared the clinical samples. H.V.W.S. and W.D. helped write the manuscript. D.X, C.FN, J. Y.‐C.T. supervised the process of the study and revised the manuscript.

## Supporting information



Supporting Information

## Data Availability

The data that support the findings of this study are available from the corresponding author upon reasonable request.
