# Supplementary material for: Patient‐Derived Bladder Cancer Organoids as a Valuable Tool for Understanding Tumor Biology and Developing Personalized Treatment
Source: Adv Sci (Weinh). 2025 Feb 7;12(13):2414558. doi: 10.1002/advs.202414558 (PMC11967763; doi:10.1002/advs.202414558)
Supplement: Supplementary file 1 — Supporting Information [file ADVS-12-2414558-s001.docx]

**Supplementary information**

**Patient-Derived Bladder Cancer Organoids as a Valuable Tool for Understanding Tumor Biology and Developing Personalized Treatment**

**Authors:**

Zhao Hongda ^1,2^, Lin Na ^3^, Ho Vincy Wing Sze ^1,2^, Liu Kang ^1,2^, Chen Xuan ^1,2^, Wu Hongwei ^1,2^, Peter Ka-Fung Chiu ^1^, Linda Huang ^4^, Zahra Dantes ^5^, Chau Ho-fai ^6^, Wu Dinglan ^1^, Ding Xiaofan ^3 *^, Chi Fai Ng ^1 *^, and Jeremy Yuen-Chun Teoh ^1,2,7 *^.

**Supplementary figures**

**Figure S1.** Representative bright field images of bladder cancer PDOs

**
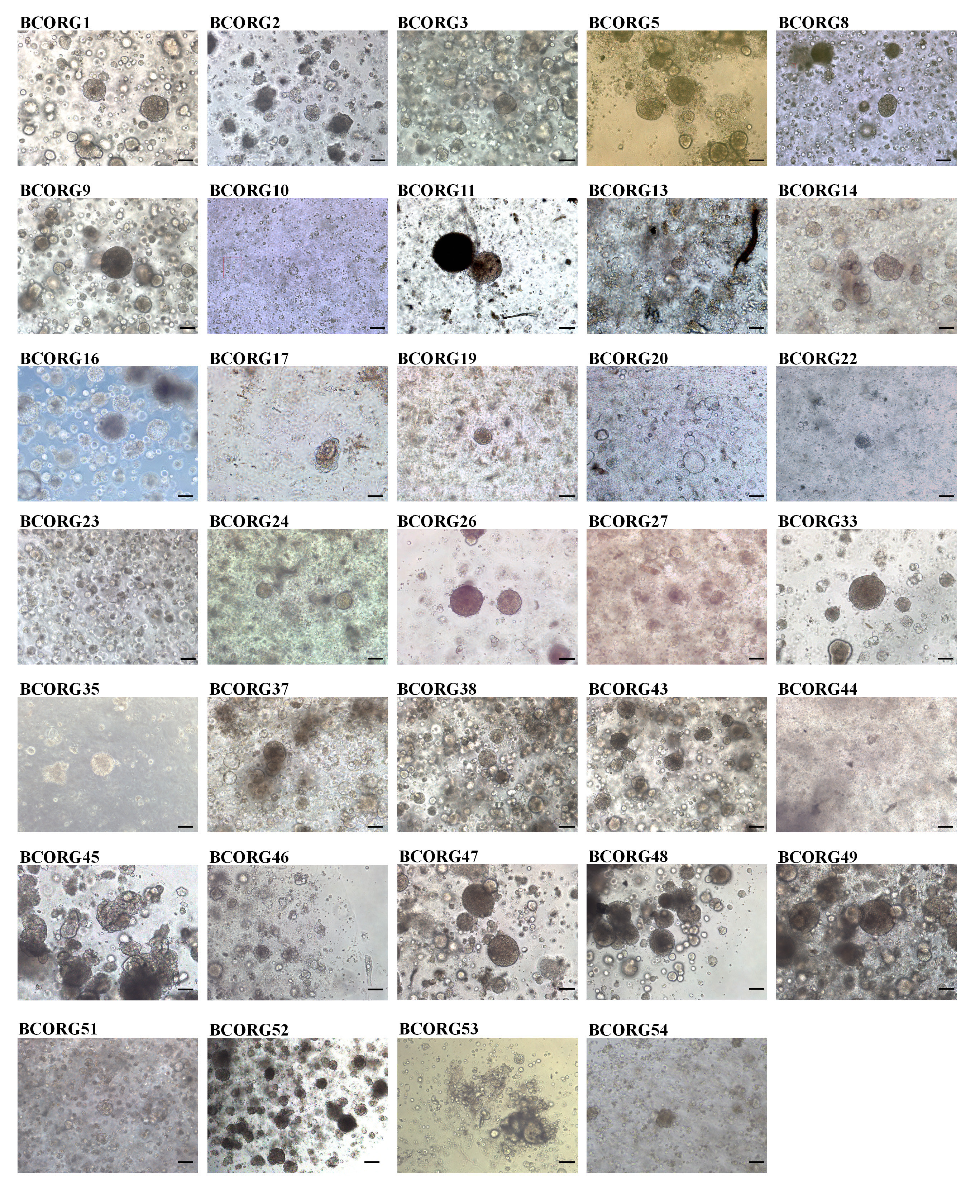
**

**Figure S2.** H&E staining and IHC staining of parental tumors for indicated markers and whole mount IF staining of PDOs for indicated markers.


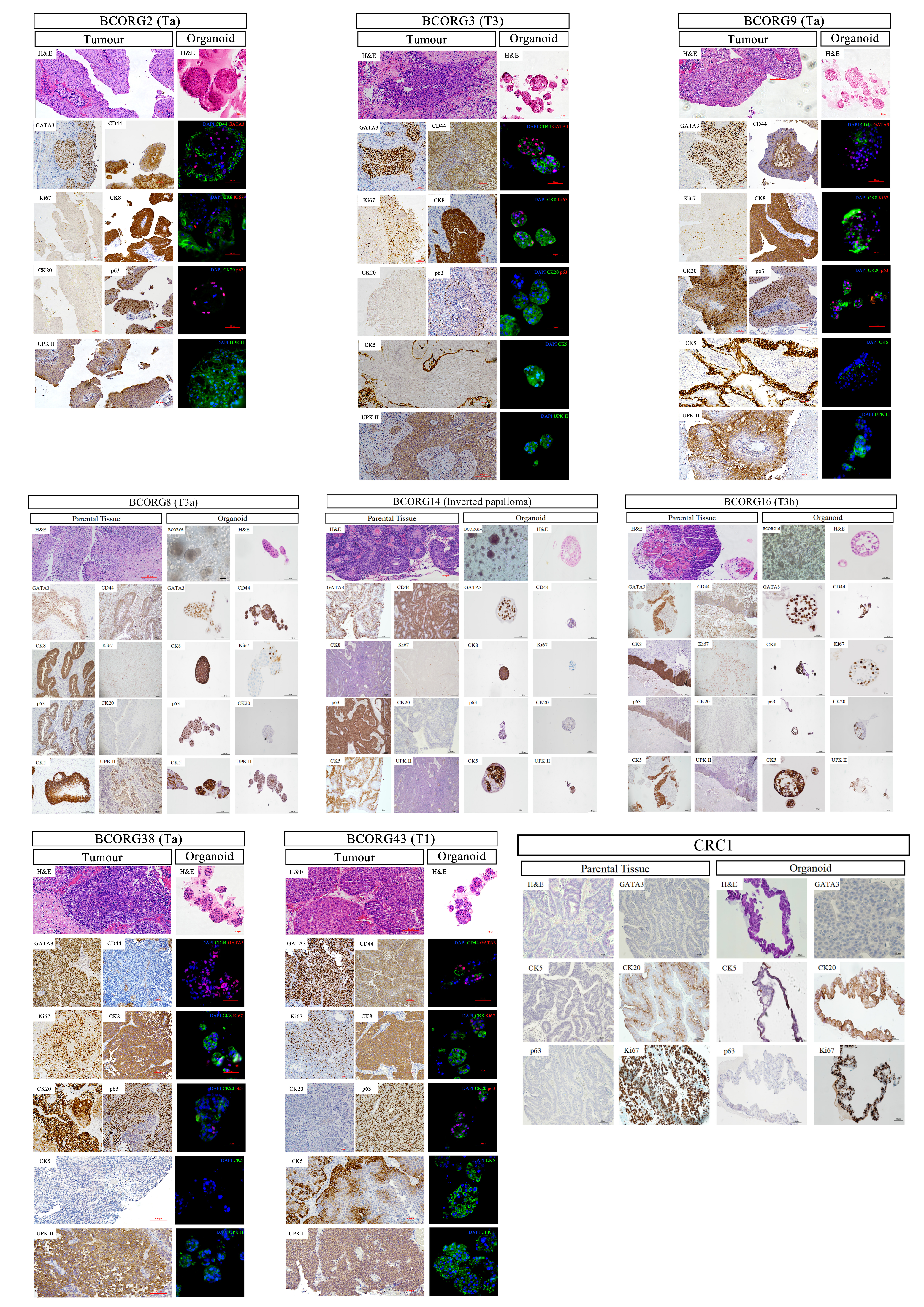


**Figure S3.** Corrected allelic fraction of all shared and private SNVs in PDOs and parental tumors.

**
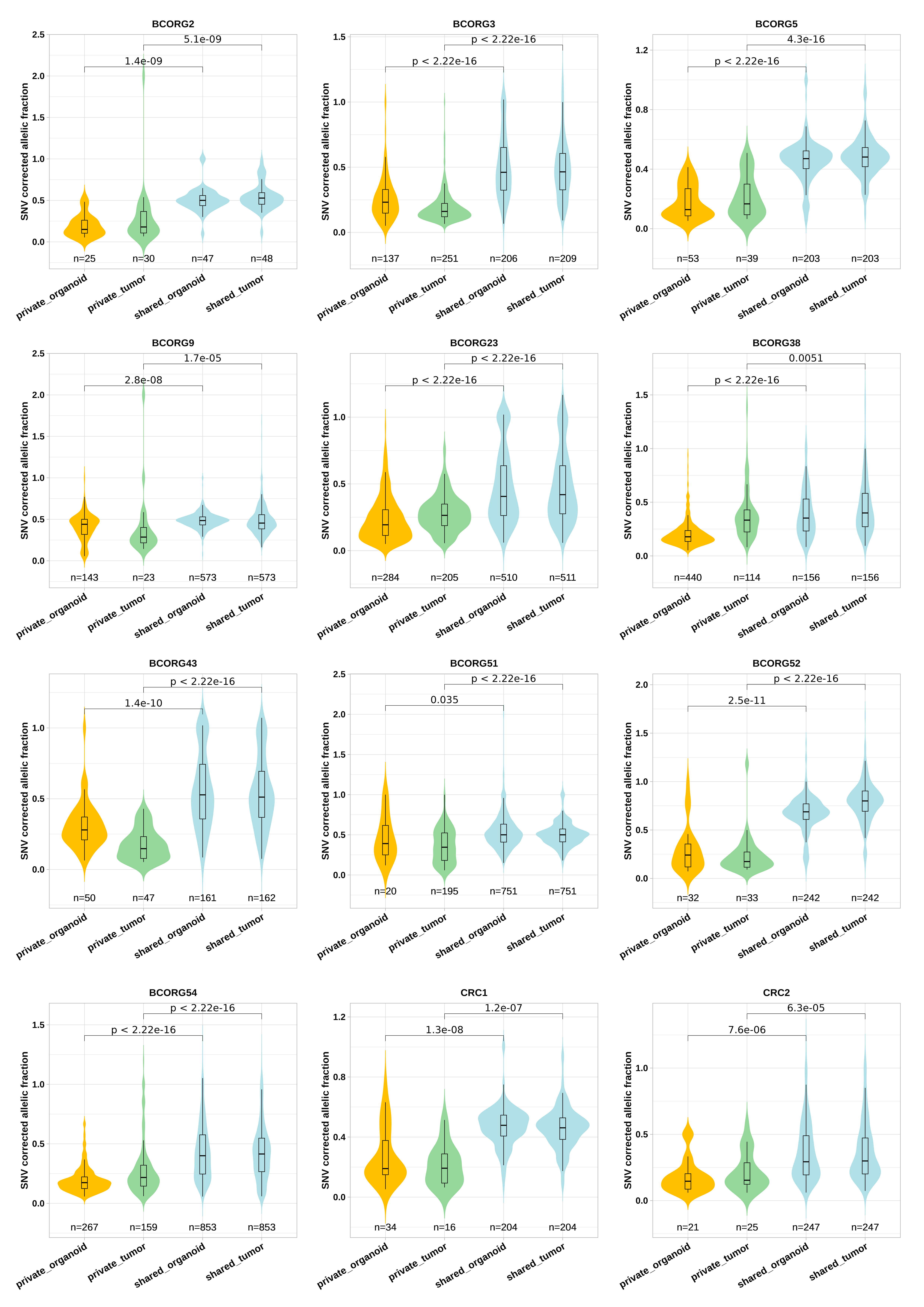
**

**Figure S4.** Copy-number and point mutations profiles between PDOs and parental tumors.


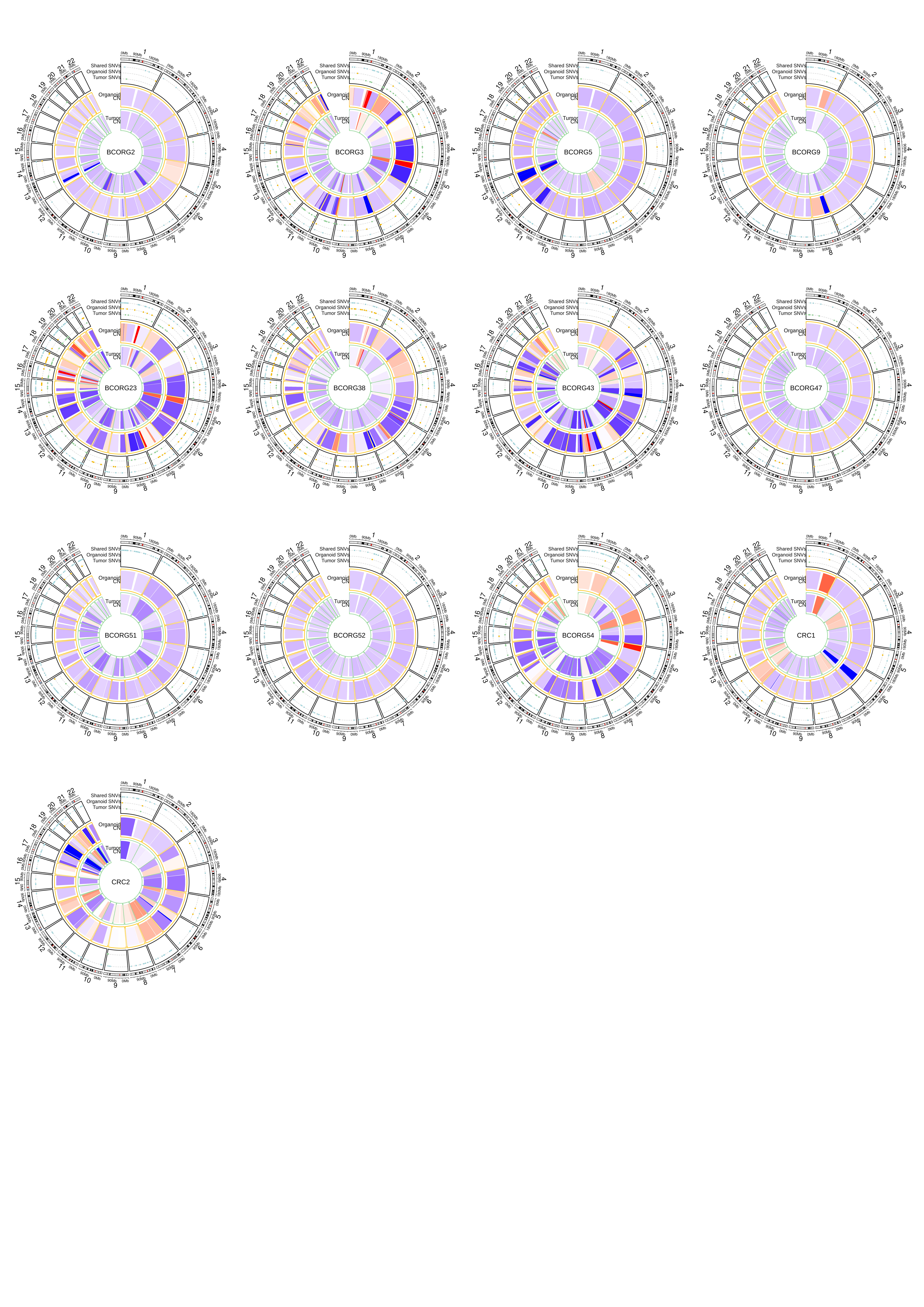


**Figure S5.** RNA similarity between matched PDOs and parental tumors

**
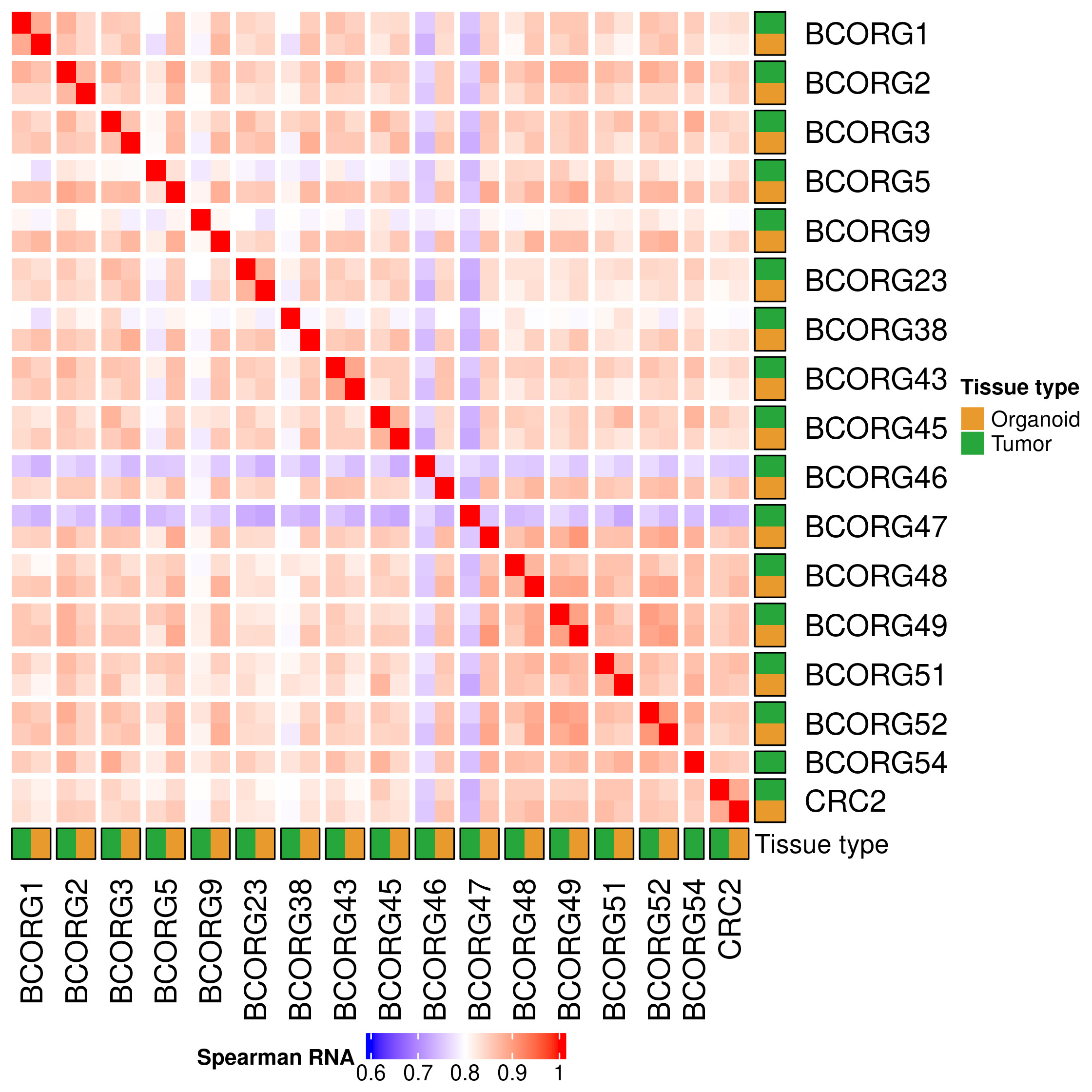
**

**Figure S6.** Effects on the viability of chemotherapy or targeted drug using an organoid-formation assay. Red square, response; no square, no response. Scale bars, 100 mm.

**
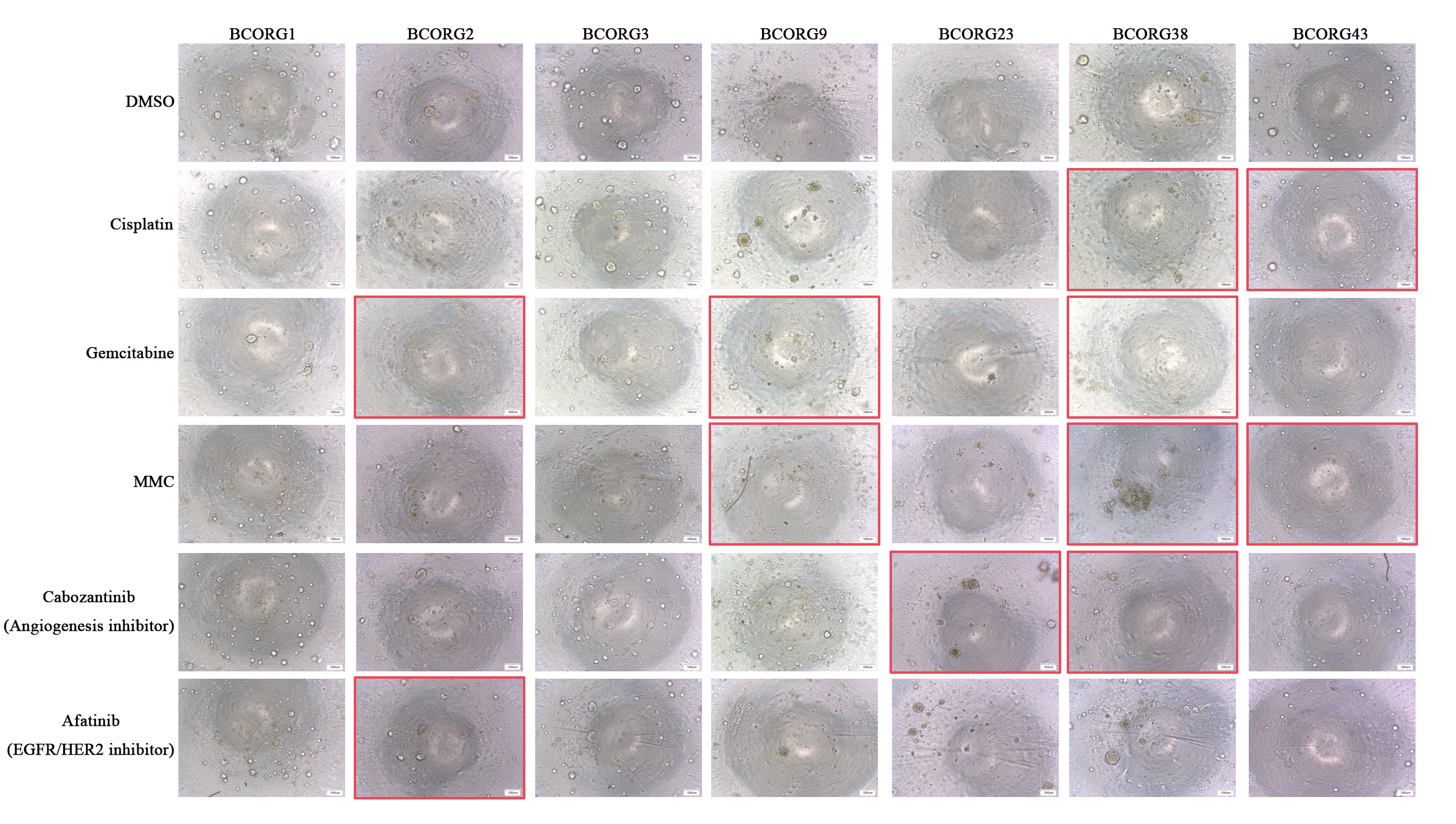
**

**Figure S7.** H&E and IHC staining of xenograft generated from organoids. (Scale bar, 50um)


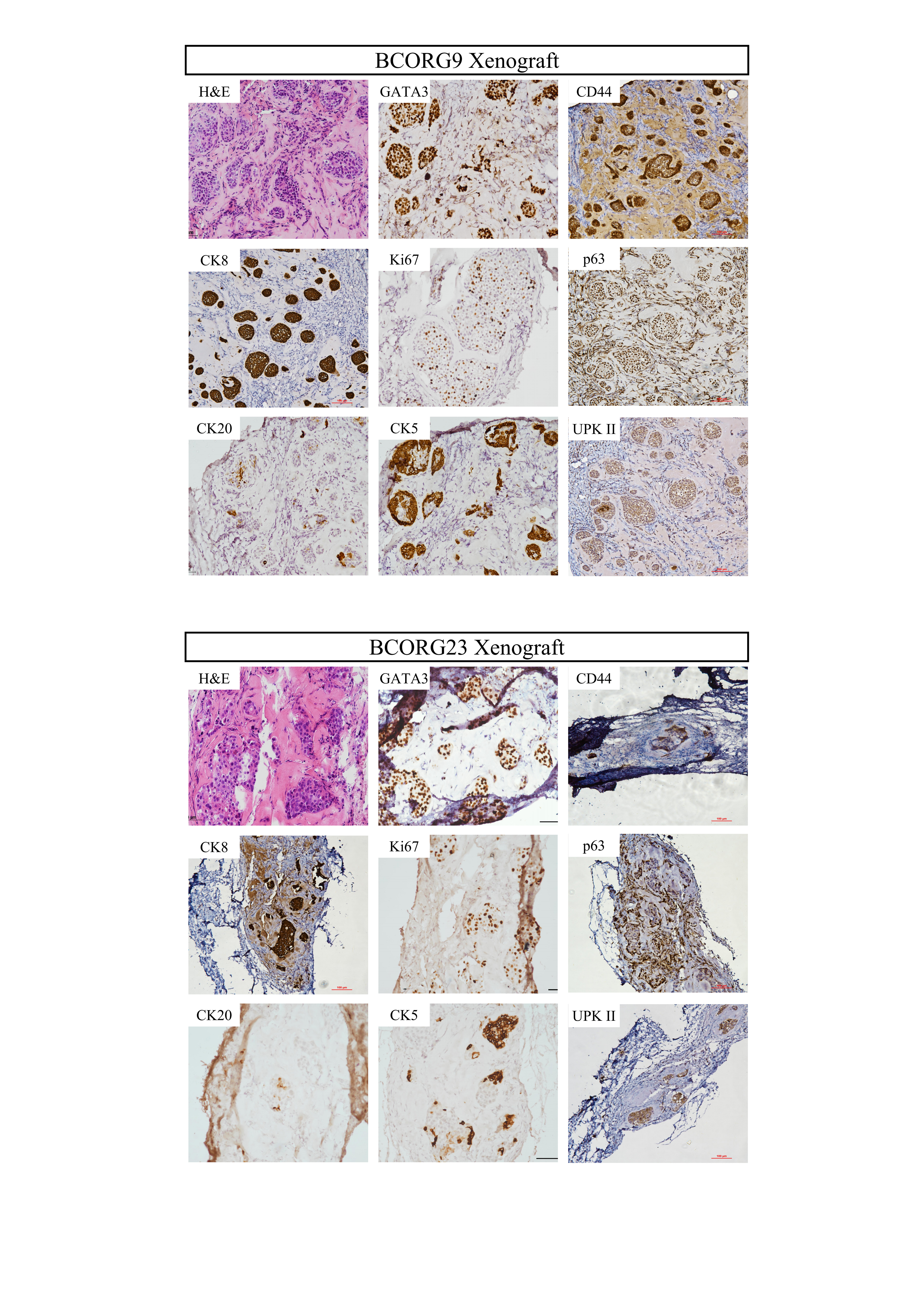


**Figure S8.** Four PDOs were treated with EPZ-011989 or vehicle control for 5 days before they were subjected to CellTiter-Glo 3D assay (n=4). IC50 were analysed by GraphPad Prism 9 software with nonlinear regression (curve fit) and the equation log(inhibitor) versus normalized response. Error bars represent ± SEM.

**
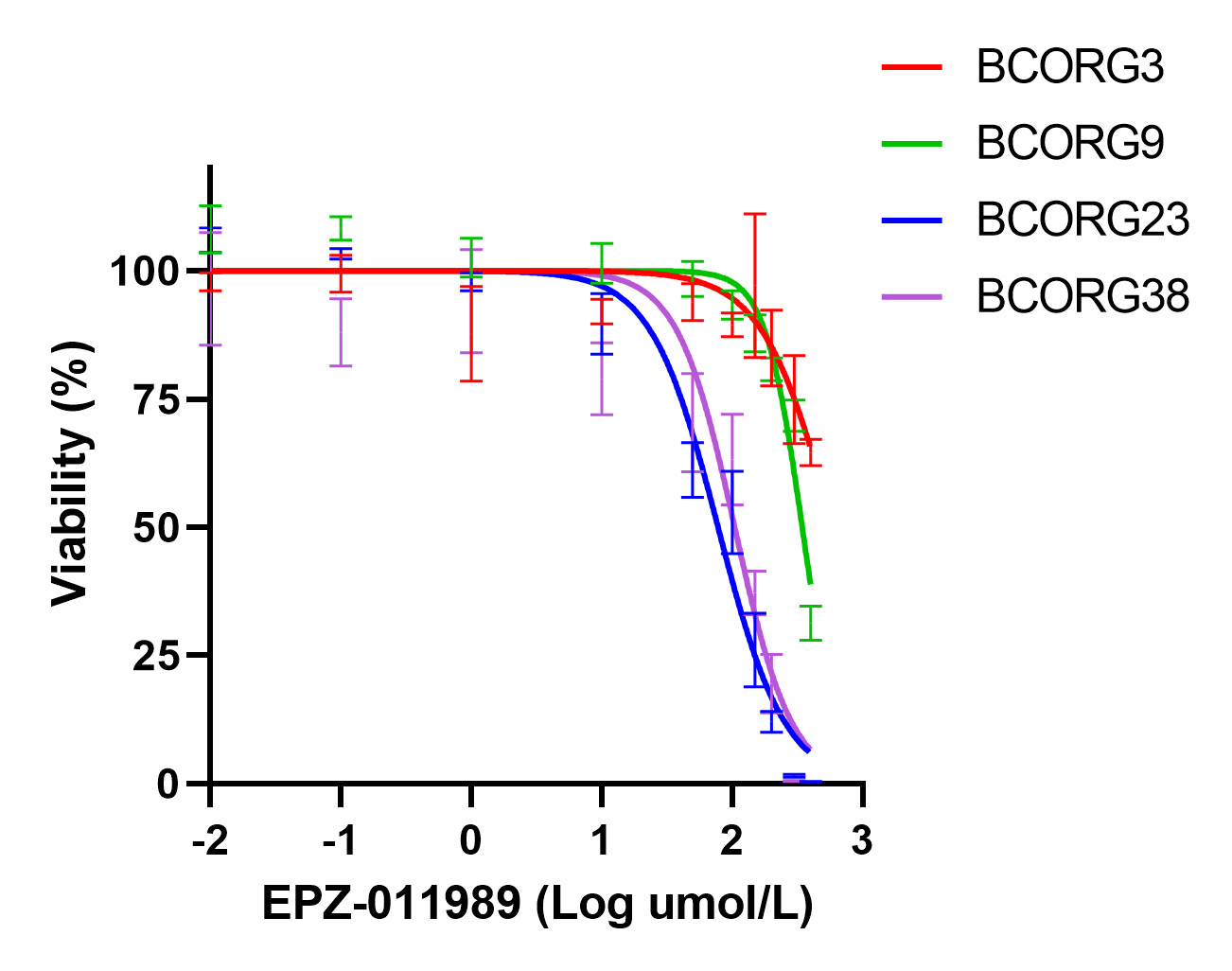
**

**Figure S9.** Cell surface expression level of MHC-I in EZH2i-treated organoid cells (EPZ-011989 5uM) were analyzed by immunofluorescence after 10 days of treatment.


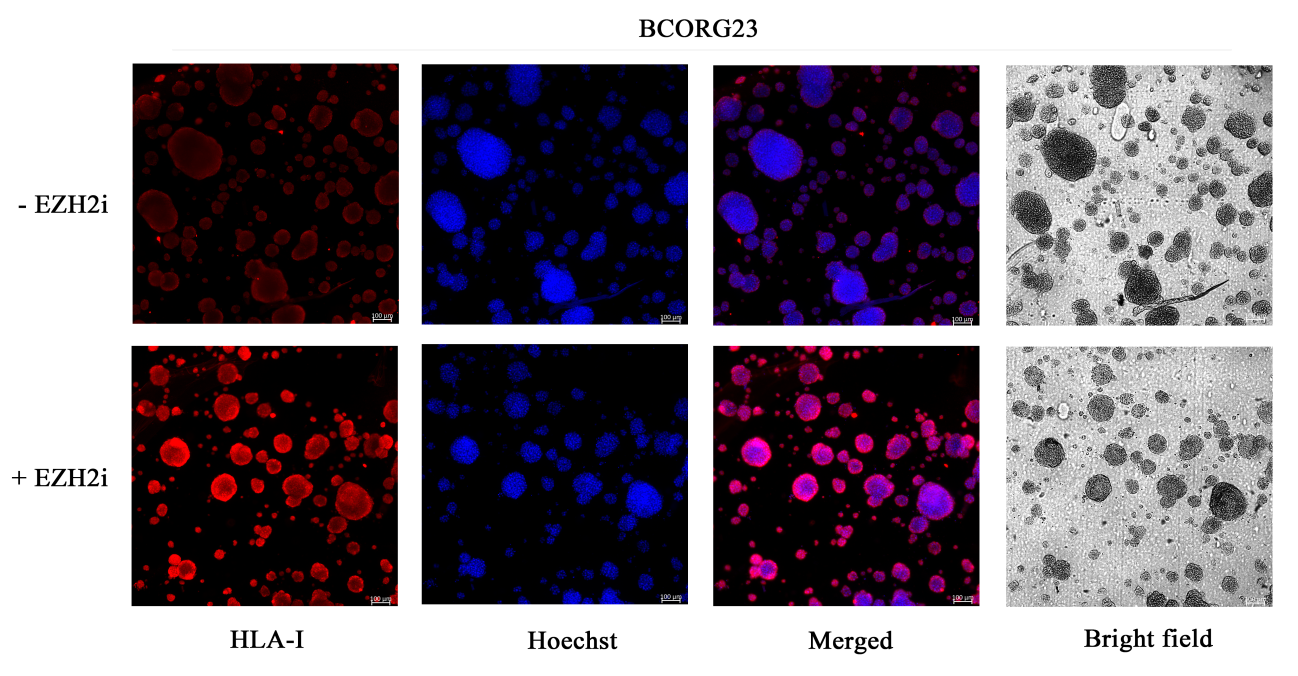

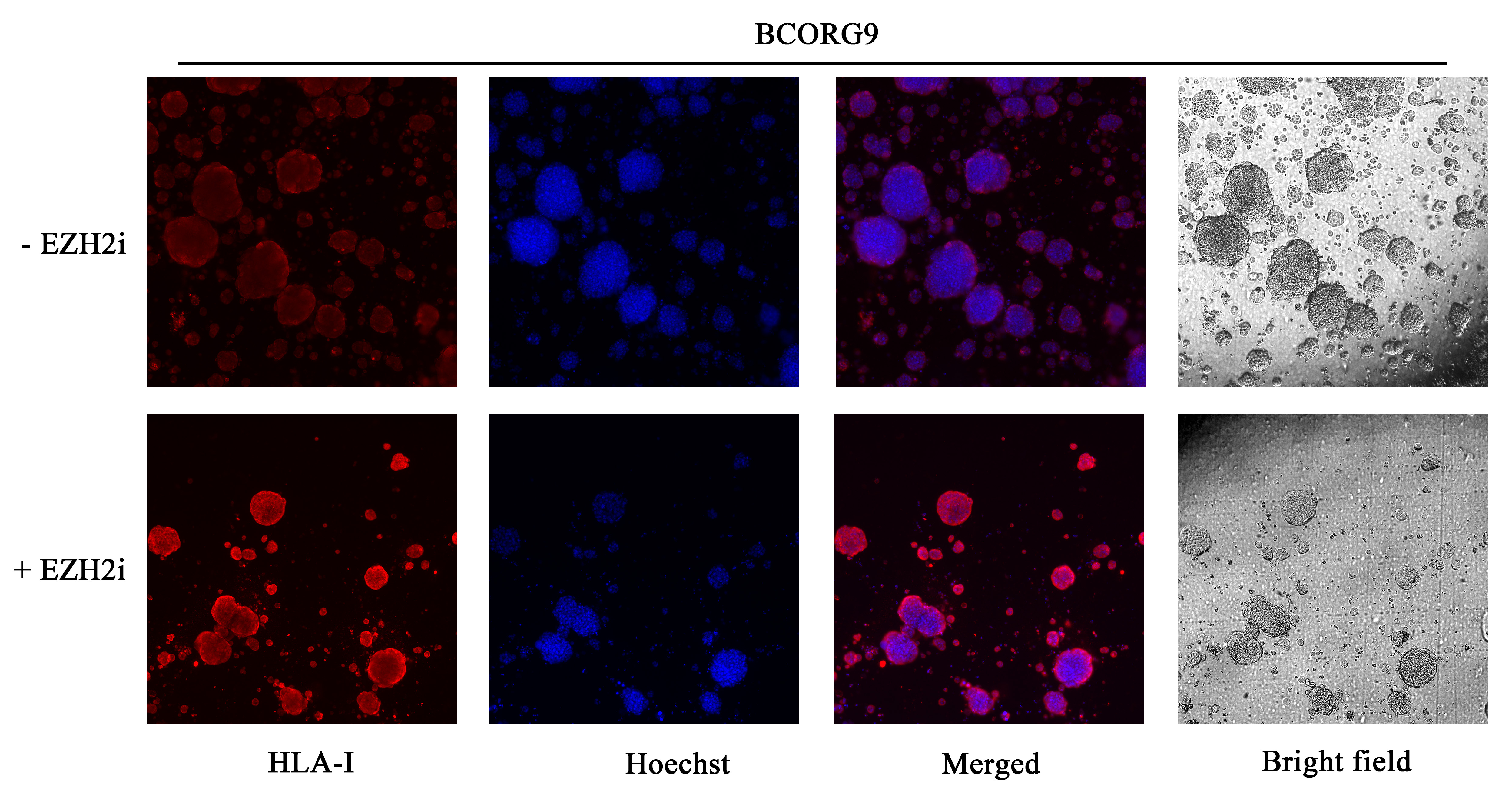


**Figure S10**. After 72 hours of co-culture, the concentration of IFN-y in the supernatant was analyzed by ELISA. The statistical differences between groups were analyzed using unpaired two-tailed t-test. Error bars represent ± SEM. *p < 0.05; **p < 0.01; ***p < 0.001.


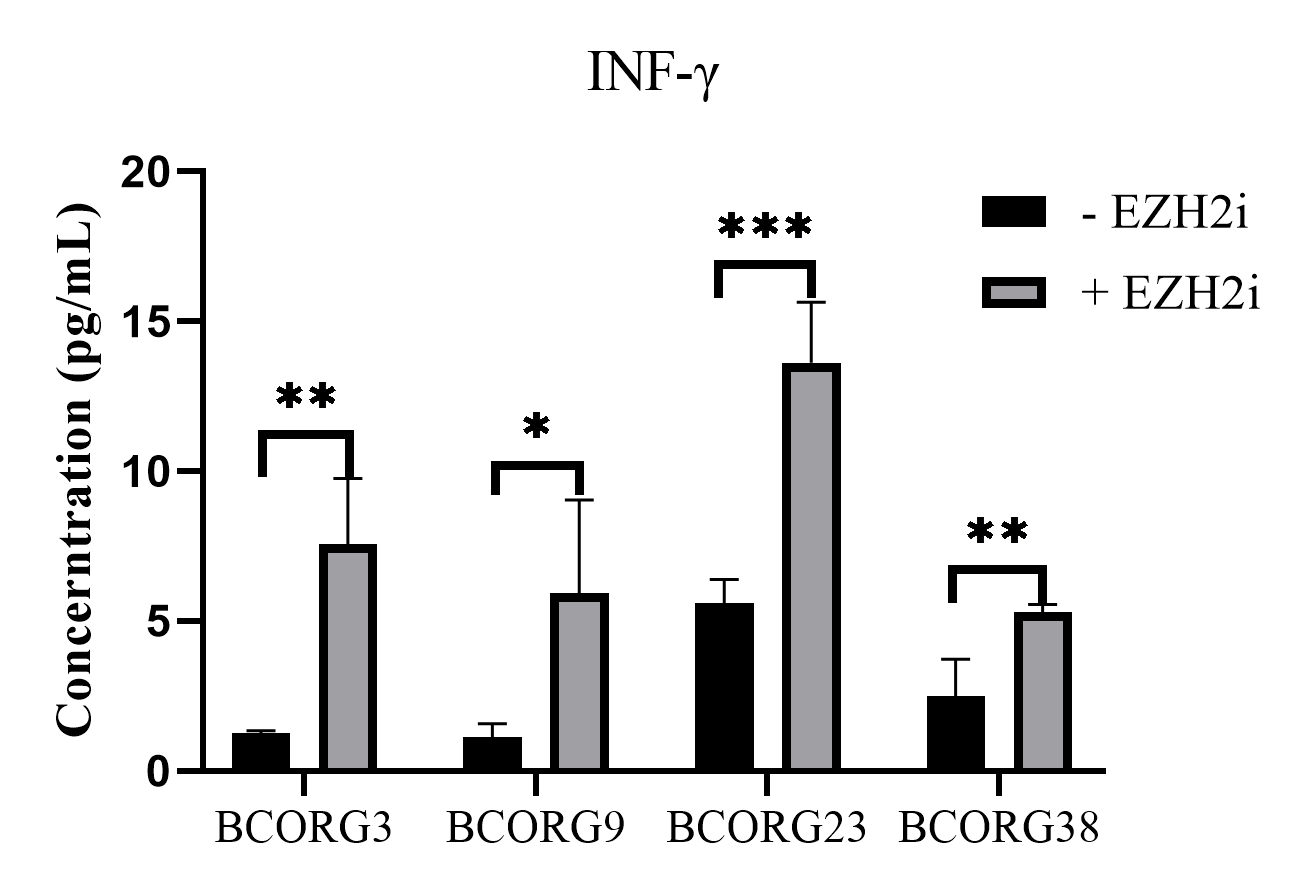


**Figure S11**. Microphotographs of BCORG3 organoids after 24 h co-culture with autologous tumor-reactive T cells. Organoids were labelled with CellTracker Red prior to co-culture.

**
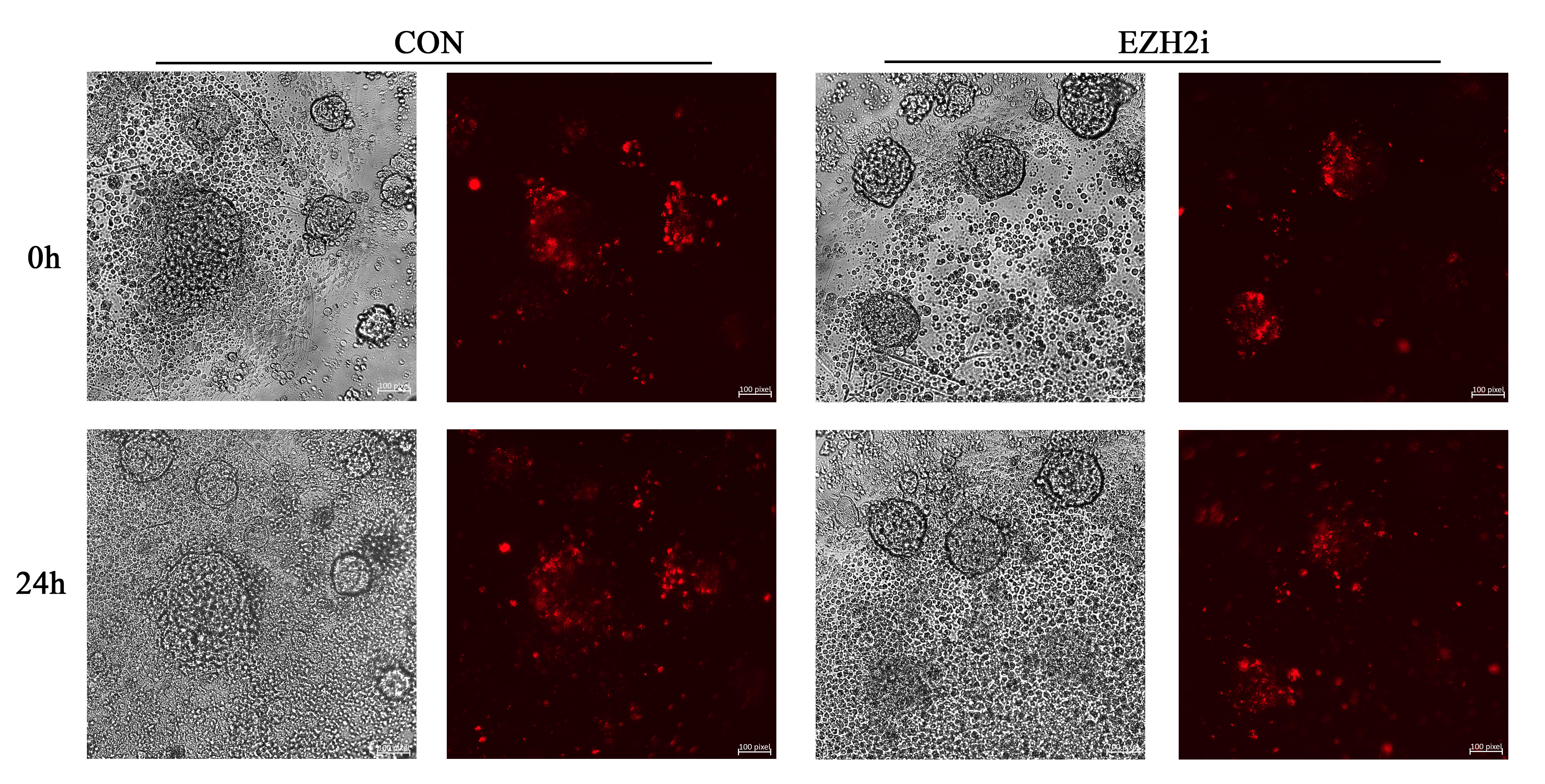
**

**Figure S12.** Microphotographs of BCORG23 organoids after 24 hr co-culture with autologous tumor-reactive T cells.


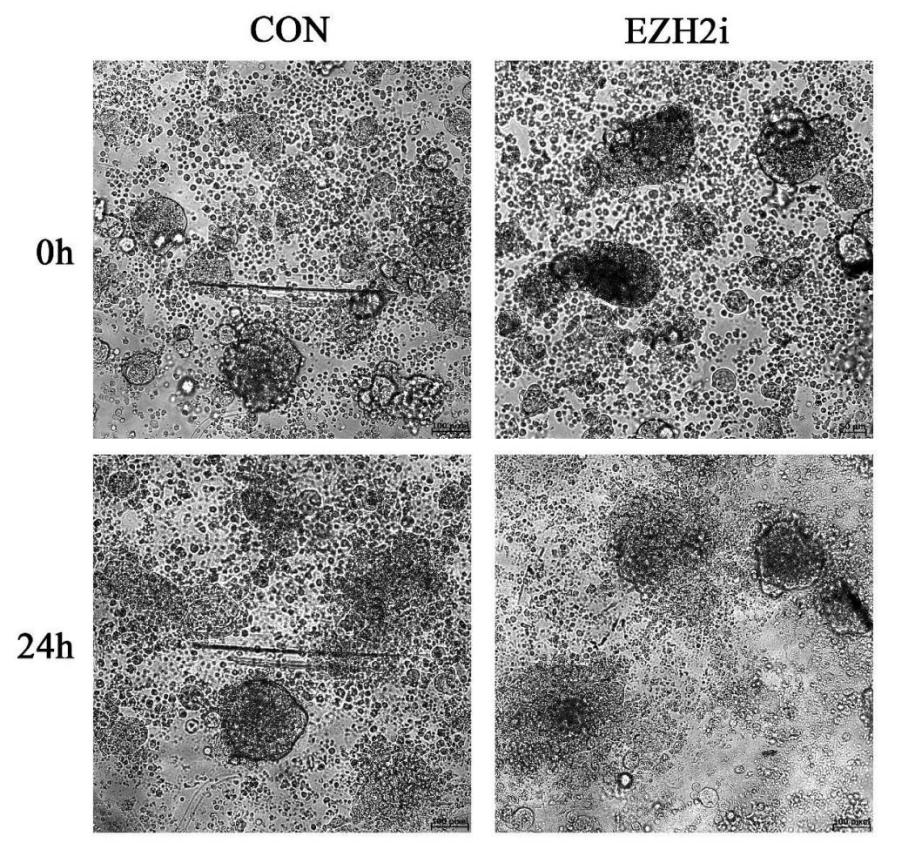


**Supplementary Tables**

**Table S1.** Summary of clinical information for 56 bladder tumor cases

| **Line** | **Age** | **Gender** | **Smoking status** | **Operation** | **Tumor Stage** | **Tumor Grade** | **Concomitant CIS** | **LVI** | **Variant of histology** | **Postoperative threatment** | **Tumor Recurrence** | **Tumor Progression** |
| --- | --- | --- | --- | --- | --- | --- | --- | --- | --- | --- | --- | --- |
| BCORG1 | 66 | Male | Ex-smoker | TURBT + cystectomy | T1N2 | Low Grade | No | Yes |  | G/C + Pemb + T/C | Yes | Yes |
| BCORG2 | 70 | Male | Nonsmoker | TURBT | Ta | Low Grade | No | No |  | No | Yes | No |
| BCORG3 | 64 | Male | Current smoker | TURBT + cystectomy | T3 | High Grade | No | No |  | G/C | No | No |
| BCORG4 | 73 | Male | Ex-smoker | TURBT | Ta | High Grade | No | No |  | No | No | No |
| BCORG5 | 69 | Male | Ex-smoker | TURBT | Ta | High Grade | No | No |  | No | Yes | No |
| BCORG6 | 64 | Male | Current smoker | Radical cystectomy | T3a | High Grade | No | No |  | G/C | No | No |
| BCORG7 | 85 | Male | Nonsmoker | TURBT | T1 | High Grade | No | No |  | BCG | Yes | No |
| BCORG8 | 68 | Female | Current smoker | Radical cystectomy | T3a | High Grade | No | Yes |  | No | No | No |
| BCORG9 | 81 | Male | Nonsmoker | TURBT | Ta | Low Grade | No | No |  | No | No | No |
| BCORG10 | 88 | Female | Nonsmoker | TURBT | Ta | Low Grade | No | No |  | No | Yes | Yes |
| BCORG11 | 89 | Male | Current smoker | TURBT | T1 | High Grade | No | No |  | No | No | No |
| BCORG12 | 70 | Male | Nonsmoker | TURBT | Ta | High Grade | Yes | No |  | BCG | Yes | No |
| BCORG13 | 81 | Male | Ex-smoker | TURBT+NU | T1 CaB+T3 UTUC | High Grade | No | No |  | No | No | No |
| BCORG14 | 51 | Male | Nonsmoker | TURBT | N/A | N/A | No | No | Inverted papilloma | No | No | No |
| BCORG15 | 65 | Male | Ex-smoker | Radical cystectomy | T2 | High Grade | No | No |  | G/C | Yes | Yes |
| BCORG16 | 76 | Male | Current smoker | TURBT + cystectomy | T3b | High Grade | No | N/A |  | No | No | No |
| BCORG17 | 77 | Male | Current smoker | TURBT | Ta | Low Grade | No | No |  | No | Yes | No |
| BCORG18 | 82 | Male | Ex-smoker | Radical cystectomy | T3a | High Grade | No | Yes |  | No | Yes | Yes |
| BCORG19 | 65 | Male | N/A | TURBT | T1 | High Grade | No | No |  | No | Yes | Yes |
| **Line** | **Age** | **Gender** | **Smoking status** | **Operation** | **Tumor Stage** | **Tumor Grade** | **Concomitant CIS** | **LVI** | **Variant of histology** | **Postoperative threatment** | **Tumor Recurrence** | **Tumor Progression** |
| BCORG20 | 74 | Male | Ex-smoker | TURBT | Tis | N/A | Yes | No |  | BCG | No | No |
| BCORG21 | 82 | Male | Current smoker | TURBT | T2 | High Grade | No | No |  | No | No | Yes |
| BCORG22 | 95 | Female | Nonsmoker | TURBT | T1 | High Grade | No | No |  | No | Yes | No |
| BCORG23 | 76 | Male | Ex-smoker | TURBT | T2 | High Grade | No | Yes |  | MMC + 5FU | No | Yes |
| BCORG24 | 88 | Male | Ex-smoker | TURBT | Ta | Low Grade | No | No |  | No | Yes | No |
| BCORG25 | 74 | Male | Ex-smoker | Radical cystectomy | T1 | High Grade | No | No |  | No | No | No |
| BCORG26 | 80 | Male | Ex-smoker | TURBT | Ta | Low Grade | No | No |  | No | No | No |
| BCORG27 | 69 | Male | Current smoker | TURBT | T1 | High Grade | No | No |  | No | Yes | Yes |
| BCORG28 | 74 | Male | Ex-smoker | TURBT | T2 | High Grade | No | No |  | Radical cystectomy | No | No |
| BCORG29 | 72 | Male | Ex-smoker | TURBT | N/A | N/A | No | No | Adenocarcinoma | No | No | Yes |
| BCORG30 | 72 | Male | Ex-smoker | TURBT | N/A | N/A | No | No | Adenocarcinoma | No | No | Yes |
| BCORG31 | 73 | Female | Nonsmoker | TURBT | N/A | N/A | No | No | Inverted papilloma | No | No | No |
| BCORG32 | 68 | Male | Ex-smoker | TURBT | T1 | High Grade | Yes | No |  | No | No | Yes |
| BCORG33 | 82 | Male | Ex-smoker | TURBT | Ta | Low Grade | No | No |  | BCG | No | No |
| BCORG34 | 81 | Male | Current smoker | TURBT | T1 | Low Grade | No | No |  | No | No | No |
| BCORG35 | 78 | Male | Current smoker | TURBT | Ta | Low Grade | No | No |  | No | Yes | Yes |
| BCORG36 | 52 | Male | Current smoker | TURBT | T4a | High Grade | Yes | Yes |  | G/C + 5-FU | Yes | Yes |
| BCORG37 | 71 | Male | Current smoker | TURBT | Ta | High Grade | No | No |  | BCG | No | No |
| BCORG38 | 67 | Male | Ex-smoker | TURBT | Ta | High Grade | No | No |  | BCG | Yes | Yes |
| BCORG39 | 89 | Female | Nonsmoker | TURBT | T3b | High Grade | No | No |  | No | No | No |
| BCORG40 | 59 | Male | Ex-smoker | TURBT | T2 | High Grade | No | No |  | No | Yes | Yes |
| **Line** | **Age** | **Gender** | **Smoking status** | **Operation** | **Tumor Stage** | **Tumor Grade** | **Concomitant CIS** | **LVI** | **Variant of histology** | **Postoperative threatment** | **Tumor Recurrence** | **Tumor Progression** |
| BCORG41 | 82 | Female | Nonsmoker | TURBT | T3a | High Grade | No | No |  | No | No | No |
| BCORG42 | 68 | Male | N/A | TURBT | Ta | High Grade | No | No |  | No | No | No |
| BCORG43 | 71 | Male | N/A | TURBT | T1 | High Grade | No | No |  | BCG | Yes | Yes |
| BCORG44 | 56 | Female | N/A | TURBT | T2 | High Grade | No | No |  | G/C + Pemb | No | No |
| BCORG45 | 75 | Male | Nonsmoker | TURBT | T1 | High Grade | Yes | No | Glandular differentiation | No | Yes | Yes |
| BCORG46 | 71 | Female | Nonsmoker | TURBT | Ta | High Grade | No | No |  | No | No | No |
| BCORG47 | 77 | Female | Nonsmoker | TURBT | Ta | Low Grade | No | No |  | No | No | No |
| BCORG48 | 74 | Male | Ex-smoker | TURBT | Ta | High Grade | No | No |  | No | No | No |
| BCORG49 | 66 | Male | Ex-smoker | TURBT | Ta | Low Grade | No | No |  | No | No | No |
| BCORG50 | 70 | Male | Ex-smoker | Radical cystectomy | T2 | High Grade | No | No |  | No | No | No |
| BCORG51 | 71 | Female | N/A | TURBT | Ta | High Grade | No | No |  | No | No | No |
| BCORG52 | 72 | Male | Current smoker | TURBT | Ta | Low Grade | No | No |  | No | No | No |
| BCORG53 | 96 | Male | Nonsmoker | TURBT | Ta | Low Grade | No | No |  | No | No | No |
| BCORG54 | 64 | Female | Nonsmoker | TURBT | T1 | High Grade | No | No |  | No | No | No |
| CRC1 | 71 | Male | Nonsmoker | TURBT | N/A | N/A | No | No | Metastatic adenocarcinoma from colon cancer | No | No | Yes |
| CRC2 | 75 | Male | N/A | TURBT | N/A | N/A | No | No | Metastatic adenocarcinoma from colon cancer | No | No | No |
| BCG= Bacillus Calmette-Guerin; G/C= gemcitabine/cisplatin; MMC=mitomycin; Pemb=Pembrolizumab; T/C=paclitaxel/carboplatin; 5-FU= 5-Fluorouracil  N/A= not available; CIS or Tis= carcinoma in situ; NU= nephroureterectomy; TURBT= Trans urethral removal of bladder tumor; UTUC= upper tract urothelial carcinoma. | | | | | | | | | | | | |

**Table S2**. Summary of basic information for bladder cancer PDOs.

| Line | Passage number | Main PDO morphology | Xenograft formation |
| --- | --- | --- | --- |
| BCORG1 | 39 | Solid | Yes |
| BCORG2 | 22 | Mixed | N/D |
| BCORG3 | 43 | Solid | N/D |
| BCORG5 | 4 | Solid | N/D |
| BCORG8 | 2 | Solid | N/D |
| BCORG9 | 45 | Solid | Yes |
| BCORG10 | 1 | Mixed | N/D |
| BCORG11 | 3 | Solid | N/D |
| BCORG13 | 3 | Solid | N/D |
| BCORG14 | 7 | Solid | N/D |
| BCORG16 | 9 | Mixed | N/D |
| BCORG17 | 1 | Solid | N/D |
| BCORG19 | 2 | Solid | N/D |
| BCORG20 | 1 | Hollow | N/D |
| BCORG22 | 3 | Mixed | N/D |
| BCORG23 | 46 | Mixed | Yes |
| BCORG24 | 2 | Solid | N/D |
| BCORG26 | 5 | Mixed | N/D |
| BCORG27 | 3 | Mixed | N/D |
| BCORG33 | 3 | Mixed | N/D |
| BCORG35 | 2 | Mixed | N/D |
| BCORG37 | 4 | Mixed | N/D |
| BCORG38 | 46 | Solid | No |
| BCORG43 | 30 | Solid | N/D |
| BCORG44 | 2 | Solid | N/D |
| BCORG45 | 25 | Mixed | Yes |
| BCORG46 | 10 | Solid | N/D |
| BCORG47 | 4 | Solid | N/D |
| BCORG48 | 15 | Solid | Yes |
| BCORG49 | 2 | Mixed | N/D |
| BCORG51 | 1 | Mixed | N/D |
| BCORG52 | 9 | Solid | N/D |
| BCORG53 | 1 | Mixed | N/D |
| BCORG54 | 1 | Mixed | N/D |
| N/D= not done | | | |

**Table S3.** Drug list and working concentrations.

| **No.** | **Drug Name** | **Concentration (uM)** |
| --- | --- | --- |
| 1 | 5-Fluorouracil | 426 |
| 2 | Afatinib | 0.052 |
| 3 | Cabazitaxel | 0.27 |
| 4 | Cabozantinib | 4.61 |
| 5 | Carboplatin | 135 |
| 6 | Cisplatin | 14.4 |
| 7 | Crizotinib | 0.913 |
| 8 | Cyclophosphamide (Mafosfamide) | 81.9 |
| 9 | Docetaxel | 5.47 |
| 10 | Doxorubicin | 2.75 |
| 11 | Epirubicin | 1.73 |
| 12 | Erdafitinib | 3.133 |
| 13 | Erlotinib | 3.15 |
| 14 | Everolimus | 0.064 |
| 15 | Gefitinib | 0.356 |
| 16 | Gemcitabine | 89.3 |
| 17 | Lapatinib | 4.18 |
| 18 | Methotrexate | 1.31 |
| 19 | Mitomycin C | 2.18 |
| 20 | Olaparib (SN-38) | 13.1 |
| 21 | Paclitaxel | 4.27 |
| 22 | Pazopanib | 133 |
| 23 | Sunitinib | 0.181 |
| 24 | Temsirolimus | 0.568 |
| 25 | Vinblastine | 0.035 |
| 26 | Vinflunine | 15 |

**Table S4.** Drug test results of PDOs to single chemotherapeutic drugs, related to Figure 6.

| Drug | Sample ID | Normalized AUC | LogIC50 |
| --- | --- | --- | --- |
| Cisplatine | BCORG1 | 0.781 | 1.744 |
|  | BCORG2 | 0.594 | 0.851 |
|  | BCORG3 | 0.770 | 1.838 |
|  | BCORG9 | 0.5997 | 0.967 |
|  | BCORG23 | 0.665 | 1.414 |
|  | BCORG38 | 0.135 | -2.947 |
|  | BCORG43 | 0.558 | 0.842 |
|  | BCORG45 | 0.552 | 0.709 |
|  | BCORG46 | 0.842 | 2.023 |
|  | BCORG48 | 0.863 | 2.085 |
|  | BCORG49 | 0.368 | -0.558 |
|  | BCORG52 | 0.464 | 0.197 |

| Drug | Sample ID | Normalized AUC | LogIC50 |
| --- | --- | --- | --- |
| Gemcitabine | BCORG1 | 0.620 | 1.3 |
|  | BCORG2 | 0.1097 | -3.83 |
|  | BCORG3 | 0.751 | 2.826 |
|  | BCORG9 | 0.488 | 0.450 |
|  | BCORG23 | 0.714 | 1.834 |
|  | BCORG38 | 0.066 | -3.457 |
|  | BCORG43 | 0.5598 | 1.226 |
|  | BCORG45 | 0.177 | -2.937 |
|  | BCORG46 | 0.571 | 1.08 |
|  | BCORG48 | 0.634 | 1.787 |
|  | BCORG49 | 0.593 | 1.165 |
|  | BCORG52 | 0.482 | 0.325 |
